# Metabolic Profiling and Quantitative Analysis of Cerebrospinal Fluid Using Gas Chromatography–Mass Spectrometry: Current Methods and Future Perspectives

**DOI:** 10.3390/molecules26123597

**Published:** 2021-06-11

**Authors:** Alisa Pautova, Natalia Burnakova, Alexander Revelsky

**Affiliations:** 1 Federal Research and Clinical Center of Intensive Care Medicine and Rehabilitology, Laboratory of Human Metabolism in Critical States, Negovsky Research Institute of General Reanimatology, Petrovka str. 25-2, 107031 Moscow, Russia; 2 Laboratory of Mass Spectrometry, Chemistry Department, Lomonosov Moscow State University, GSP-1, Leninskie Gory, 1-3, 119991 Moscow, Russia; nburnakova@me.com (N.B.); sorbent@yandex.ru (A.R.)

**Keywords:** metabolomics, targeted analysis, nontargeted analysis, sample preparation, derivatization, validation, biomarkers

## Abstract

Cerebrospinal fluid is a key biological fluid for the investigation of new potential biomarkers of central nervous system diseases. Gas chromatography coupled to mass-selective detectors can be used for this investigation at the stages of metabolic profiling and method development. Different sample preparation conditions, including extraction and derivatization, can be applied for the analysis of the most of low-molecular-weight compounds of the cerebrospinal fluid, including metabolites of tryptophan, arachidonic acid, glucose; amino, polyunsaturated fatty and other organic acids; neuroactive steroids; drugs; and toxic metabolites. The literature data analysis revealed the absence of fully validated methods for cerebrospinal fluid analysis, and it presents opportunities for scientists to develop and validate analytical protocols using modern sample preparation techniques, such as microextraction by packed sorbent, dispersive liquid–liquid microextraction, and other potentially applicable techniques.

## 1. Introduction

Modern differential diagnostics of a wide variety of diseases and pathologies is not complete without analyzing the composition of biological fluids of the body. The most available and studied fluids are blood and urine. Less studied, as well as less available, is cerebrospinal fluid (CSF). CSF performs a number of important physiological functions in the brain and spinal cord, providing metabolic processes between the blood and the brain. Chemical compounds of different structures can penetrate from the blood through the blood–brain barrier (BBB) into the CSF, and then into the brain cells, subsequently affecting the functioning of the central nervous system (CNS) [1].

The routine laboratory study of the CSF composition is usually aimed at the diagnosis of acute infectious diseases of the CNS such as meningitis and encephalitis. The current interest in the study of the CSF composition is due to the rapidly increasing number of neurodegenerative, mental, and other slowly progressive—and in most cases, incurable—diseases. Despite the advances in science and medicine, treatment of these diseases is directed at reducing the symptoms, but not at eliminating the cause of the disease, since the etiologies of most of the common diseases, such as multiple sclerosis [2,3,4,5,6,7], Parkinson’s disease [8], Alzheimer’s disease [9,10,11,12,13,14], and others, remain not fully understood.

One of the modern concepts in the medical community is the concept of “microbiota–gut–brain” connection. Previously, it was thought that the brain affects the functioning of the body “unidirectionally”, but recent research studies clearly indicate that the composition and function of the gut microbiota have an equal impact on the brain and the CNS. Numerous studies have shown that the composition of the microbiota in healthy volunteers differs from that in patients with various mental, neurodegenerative, and chronic diseases [15,16]. The gut microbiota synthesize and utilize a large number of biologically active substances, including fatty acids, amino acids, neurotransmitters, and others that can penetrate the BBB; thus, the microbiota are becoming a new potential target for the monitoring and treatment of various CNS diseases [17,18,19].

The main fact that limits the active study of the composition of the CSF is its inaccessibility. A lumbar puncture is required to obtain the CSF that carries the risk of side complications [20]. The most common one is a postdural puncture syndrome, which is accompanied by headaches and symptoms of meningism, as well as less common complications (spinal or epidural bleeding, adhesive arachnoiditis, and trauma to the spinal cord) [21]. The technique of lumbar puncture requires professionalism and experience from the specialist who performs it; this determines its nonprevalence along with the above-mentioned side effects. Lumbar puncture for diagnosis of subarachnoid hemorrhage, hydrocephalus, or infectious diseases of the CNS is frequently carried out in cases in which computed tomography or magnetic resonance imaging is not possible (with the exception of meningitis) [20].

During the diagnostic lumbar puncture, the volume of the sampled CSF is 2.0–5.0 mL (protocol approved by the regional guidelines approved by the Ministry of Health of the Russian Federation, 4 March 2004, #4.2.1887-04). Subsequent routine analysis includes the determination of cytosis, microbiological studies, and biochemical analysis (determination of the level of glucose, chlorides, etc.). Since the total volume of the CSF in an adult is 125–150 mL, any actions aimed at the additional CSF collection or procedure must be justified and approved by the local ethics committee of the institution. For scientific purposes, carrying the CSF analysis out in the residual volume of the sample after clinical and laboratory studies, which is usually about 0.5–1 mL, is highly desirable. Despite all mentioned limitations, the investigation of the CSF composition—in particular, of that of the healthy donors—is an important and crucial issue for the discovering of the new biomarkers.

The limited available sample volume requires the use of modern efficient sample preparation techniques and sensitive analytical methods for the determination of metabolites, most often at the trace level. Such methods are chromatographic ones, namely gas (GC) and high-performance liquid chromatography (HPLC) in combination with different mass-selective (MS) detectors (single quadrupole (Q), time-of-flight (TOF), including high resolution equipment and tandem MS/MS or Q-TOF) [22].

Nontargeted metabolic profiling analysis is the initial stage in the search for metabolites that distinguish patients with pathology from patients of the control group/healthy volunteers. The most common task is to identify as many chromatographic peaks in the sample as possible using the total ion current mode [8,10,23,24]. The subsequent steps should be directed at clearly establishing the formulas of the most promising markers, and statistically processing the quantitative data, which will answer the question of whether the found metabolite or several metabolites are promising biomarkers [25,26,27]. An important stage is the validation of the method for determining the target components, which currently needs to be carried out in accordance with the Food and Drug Administration (FDA) or the European Medicines Agency (EMA) guidelines [28]. Since the volume of biological samples for full validation is about 40–50 mL (for plasma samples), CSF validation requirements can be reduced because of its specificity. However, validation for the CSF metabolites is justified for several compounds simultaneously.

Based on the information provided, the following requirements for the CSF analysis method can be formulated:

- the use of the minimum (less than 0.5 mL) volume of the CSF for one analysis;
- the quantitative analysis of several target components at the trace level in one analysis.

Gas chromatography is the most common method for the analysis of volatile compounds in biological samples. Correctly selected sample preparation conditions lead to the quantitative determination of different classes of chemical compounds in one chromatographic analysis, which meets the above requirements. When comparing the capabilities of GC and HPLC, the lower cost of the GC equipment and the simpler selection of the parameters of the analysis are worth noting. Despite the wider possibilities of the HPLC in the analysis of nonvolatile high-molecular-weight compounds, gas chromatography is the “gold standard” for the tasks of metabolomics—in particular, the determination of low-molecular-weight compounds [29]. Thus, the aim of this review was to analyze the original articles published since 2000 on the study of the CSF composition using GC–MS and to describe the most promising modern methods of sample preparation, which are potentially suitable for studying CSF composition. The PubMed, Science Direct, and Google Scholar database platforms were used for the search. The keywords “cerebrospinal fluid” and “gas chromatography–mass spectrometry” or “GC–MS” were used in combination in the search list.

## 2. The Human Cerebrospinal Fluid Metabolome

A large-scale study of the CSF composition (Table 1) was carried out by a group of scientists who are the creators of the Human Metabolome Database. The main publication describing the result of their research provided a link to the created resource www.csfmetabolome.ca (accessed on 11 June 2021). From a total of 308 metabolites detected, 53 were identified using nuclear magnetic resonance (NMR) spectroscopy, 41 using GC–MS, and 17 using LC–MS [22]. Later work by the same authors described 476 metabolites [30]. At the time of preparing the present review, there were 445 metabolites in the metabolite catalog, 443 of which were listed as qualitatively and quantitatively measured. The main classes of low molecular weight metabolites found in CSF are the compounds that can be determined by GC–MS, namely amino acids, fatty acids, including short-chain ones, steroids and their derivatives, hydroxy acids, dicarboxylic acids, and nucleosides. Moreover, most of the identified compounds are neurotransmitters or their metabolites [22]. As noted by the authors, despite the greater number of compounds determined by the NMR, the potential remains with GC–MS when using selective methods of sample preparation and derivatization, as well as when using TOF mass spectrometry.

The authors also noted the presence of biological variability in the concentration of metabolites between individuals in average ±50%, and in some cases ±100%. This phenomenon should be considered by researchers who study potential biomarkers in the CSF [22]. A large-scale metabolomic–proteomic investigation was directed to the study of the biological variability of the concentrations of the CSF metabolites using normal human CSF from patients undergoing routine, non-neurological surgical procedures. As a result of the metabolomic study, which included nontargeted GC–MS analysis, 93 of 108 detected metabolites were identified, including amino acids, organic and fatty acids, nucleosides, mono- and disaccharides, the biological variability of which was 15–85% (analytical variability less than 20%) [31]. Another study was devoted to the characterization of postprandial effects on the CSF metabolic profile of healthy volunteers (*n* = 9), which was performed using GC–MS/MS. Individual plots of postprandial samples of 150 CSF hydrophilic metabolites were positioned similar to the corresponding plots of preprandial samples. The postprandial effects had a far lower impact compared with interindividual variations [32]. Thus, biological interindividual variations appear to have more significant impact on the CSF metabolite profile than food intake.

Important results were obtained describing different effects of preanalytical factors on stability of the proteomic and metabolomics profiles of the CSF. These factors were a 30/120 min delayed storage after the CSF collection at room temperature as the potential delays in the clinic, storage at 4 °C as the time that samples remain in the cooled autosampler, and repeated freeze–thaw cycles. The delayed storage factor led to the increased levels of 49 metabolites, which were analyzed using nontargeted GC–MS, and explained by metabolic processes that occurred because of the remaining white blood cells. The author’s recommendations are to remove white blood cells by the CSF centrifugation immediately after collection, use liquid nitrogen for the snap-freeze of the supernatant for storage at −80°C, and avoid freeze/thaw cycles. Samples should not be left in the autosampler for more than 24 h [33].

The chemical composition of low-molecular-weight CSF metabolites justifies its study using GC–MS in case of compliance with the necessary requirements for sampling, storage, and selection of appropriate and sensitive methods of sample preparation, which will provide quantitative determination of the target components at the required level, taking their potential biological variability among individuals into account.

## 3. Metabolic Profiling of the Cerebrospinal Fluid Using Advanced GC–MS Technologies

Different groups of authors have made attempts for the metabolic profiling of the CSF using more sensitive types of MS detectors than those described in Section 2 (Table 1). The most abundant types of mass analyzers are scanning single and triple-quadrupoles (MS/MS) and time-of-flight (TOF). TOF analyzers are more suitable for metabolic profiling because of their high speed, resolving power, sensitivity, and high quality of identification achieved by retention time, combination of accurate mass, and isotopic distribution. At the same time, the sample preparation approach for the metabolic profiling remains nonselective and includes liquid–liquid extraction (most often using methanol for protein precipitation and as extraction solvent) and widespread two-step derivatization using oxymation (the first step) and silylation (the second step) with different types of reagents to form the volatile derivatives. The most abundant type of the capillary column for the GC is a 30 m × 0.32 mm × 0.25 μm column with a phase of 5% phenyl/95% dimethyl polysiloxane crosslinked polymer, which is characterized by low bleed of the stationary phase, resistance to active compounds, and high temperature stability. This column is suitable for the determination of a wide range of compounds and produced as HP-5MS, TR-5MS, and under other trade names. However, more specific types of columns are also applied.

Application of two-dimensional GC–TOFMS led to the identification of 91 metabolites out of over 1200 detected. Sensitivity was achieved using cryogenic modulation, which concentrated analyte fractions transferred from the first (a 10 m × 0.18 mm I.D. Rxi-5ms column with film thickness of 0.18 µm) to the second column (a 1.5 m × 0.1 mm i.d. BPX-50 column with film thickness of 0.1 µm) [34]. The method was based on two types of capillary columns and two types of mass-selective detectors (DB-5 column (30 mm × 0.25 mm i.d., film thickness 1.0 μm) and GCMS-QP2010 Plus; a fused silica capillary column CP-SIL 8 CB low bleed/MS (30 m × 0.25 mm i.d., film thickness 0.25 μm) and GCMS-QP 2010 Ultra) for targeted and nontargeted analysis led to the detection of 61 metabolites, where 45 metabolites were identified with a nontargeted semiquantitative analysis. Sixteen metabolites involved in the tricarboxylic acid cycle, glycolysis, and amino acids were identified quantitatively: succinic acid, fumaric acid, malic acid, aconitic acid, isocitric acid, citric acid, alanine, valine, leucine, isoleucine, proline, serine, threonine, methionine, phenylalanine, and tyrosine [23].

Two groups of authors explored a similar multicomponent biomarker approach [25,26] using classical two-step derivatization and GC–TOFMS metabolite profiling coupled to a multiplex bioinformatics approach. The first research resulted in 40 identified compounds of 120 peaks; the second resulted in 85 structurally identified and quantified compounds of 962 metabolic signatures. The identified metabolites in the second publication were classified as sugars and sugar alcohols (24%), amino acids (28%), fatty acids (15%), organic acids (15%), and amines (2%) [26].

Careful attention should be paid to the results of the study, in which metabolic profiling was performed using not common configuration of GC with an orthogonal-accelerated TOFMS with atmospheric pressure chemical ionization interface. A distinctive feature of the chemical ionization (CI) is a softer ionization at the energy not exceeding 5 eV, compared to the electron ionization at 70 eV commonly used in GC–MS systems, which provides less fragmentation of the precursor ion. Evaluation of the analytical parameters (repeatability, reproducibility, linearity, and detection limits) using model solutions led to the successful determination of the 25 different compounds (valine, alanine, sarcosine, leucine, proline, isoleucine, benzoic acid, glycine, serine, threonine, methionine, aspartic acid, glutamic acid, phenylalanine, Phenyl-Gly, hippuric acid, caffeine, theophylline, lysine, tyrosine, 4-methyldopamine, dopamine, uric acid, 5-hydroxyindole-3-acetic, and nortriptyline). The applicability of this technique for the CSF metabolic profiling was demonstrated and resulted in 21 identified compounds from more than 300 detected [35].

Tandem GC–MS/MS was applied for the metabolic analysis in pediatric patients and revealed 180 metabolite derivatives in the CSF samples. The main metabolites were 2-ketoglutaric acid, pyridoxamine, tyrosine, 2-propyl-5-hydroxypentanoic acid, 1,5-anhydroglucitol, 2-aminobutiric acid, 2-ketoisocaproic acid, 4-hydroxyproline, acetylglycine, methionine, *N*-acetylserine, and serine [36]. Fatty acid analysis was performed using GC–MS/MS and resulted in identification of 20 compounds (6 saturated, 6 monounsaturated, and 8 polyunsaturated fatty acids) [10].

Metabolomic research studies are also in demand—specifically, studies in animals. A highly sensitive headspace solid-phase microextraction–GC–MS technique was successfully put into practice for the detection of 25 volatile constituents in rabbit CSF after intranasal administration of Asari Radix et Rhizoma frequently used in traditional Chinese medicine [37]. Numerous amino acids and related compounds (glycine, l-alanine, l-asparagine, l-glutamic acid, l-glutamine, l-isoleucine, l-leucine, l-lysine, l-methionine, l-phenylalanine, l-proline, l-serine, l-threonine, and *O*-phosphoethanolamine) were detected using GC–MS in the rat CSF [2]. GC–MS metabolomics profiling for the macaque CSF samples produced 663 variables across the two different groups of animals with depression, which were used in the subsequent multivariate analysis. In total, 37 metabolites responsible for discriminating these two groups were identified (propanoic acid, acetic acid, hydroxylamine, propanedioic acid, butanoic acid, proline, methanamine, glycine, isothiourea, nonanoic acid, carbamic acid, threonine, β-alanine, threitol, erythronic acid, l-aspartic acid, xylitol, ribitol, 2-keto-d-gluconic acid, 1,4-butanediamine, d-fructose, myoinositol, glucaric acid, hexadecanoic acid, scyllitol, gulose, heptadecanoic acid, linolelaidic acid, trans-9-octadecenoic acid, oleic acid, octadecanoic acid, *N*-acetyl-d-glucosamine, d-glycero-d-galactoheptitol, galactitol, 5-phenylvaleric acid, benzeneacetic acid, and 1*H*-indole-2-carboxylic acid) [38].

Most of the described investigations required from 25–50 µL [2,23,34,36] to 100–500 µL [25,26,37] of the CSF (even 1 mL [10]). After different steps of sample preparation, especially during derivatization and subsequent dilution with organic solvent, the final volume of the mixture is usually 100–200 µL. As the volume of CSF is usually limited, especially in cases of experimental rat or mouse models of diseases, approaches requiring extremely small amounts of the CSF and sample for the GC–MS analysis are of great interest.

Three investigations appeared to be promising in solving these issues. The analysis with modified vial design and sample workup procedure became applicable to small volume of the CSF (10 µL), and 50 µL of the final mixture was used for GC–MS analysis. The modified vial design reduced the required volume of the insert from 500 to 200 µL, and the smaller amount of derivatizing agent resulted in a reduction to 50 µL of the total volume of the mixture for the analysis. This approach had similar number of metabolites as in the analysis of >100 µL of the CSF, i.e., 73 identified compounds from 93 detected peaks, and was successfully applied for the metabolic profiling of the rat CSF [39]. Application of nontargeted metabolomics using 5 µL of the CSF for GC–TOFMS seemed to be interesting for investigation, as it resulted in 97 metabolites (including phenylalanine, leucine, threonine, valine, tryptophan, serine, glycerol, 1,5-anhydroglucitol, methionine, β-mannosylglycerate, asparagine, tyrosine, lysine, glutamine, isoleucine, proline, 2-hydroxyglutarate, tryptophan, glycine, proline, isoleucine, and alanine) being identified. Unfortunately, there was no complete information about sample preparation, particularly on derivatization and total sample volume for the analysis [41]. An analytical method based on in-liner silylation in the programmed temperature vaporizer (PTV) injector at 70 °C coupled to GC–TOFMS used only 0.01–2 µL of the CSF and was subsequently applied for metabolic profiling of the human and mouse CSF. A total of 342 peaks were found in both human and mouse profiles and 52 metabolites were identified in the human CSF (amino acids, organic acids, fatty acids, sugars, and others) [40]. The described methods for 0.01–2 and 10 µL of CSF demonstrated promising results and could be recommended for the metabolomics studies, although the method for 0.01–2 µL of CSF requires more expensive equipment (PTV injector and TOFMS) compared to those for 10 µL (GC–MS).

Metabolic profiling resulted in most cases in a number of compounds or groups of compounds that successfully distinguished the compared groups of patients or patients and healthy donors. These compounds are candidate biomarkers for a various type of diseases, including inflammatory demyelinating, neurodegenerative, oncological, infectious, mental, genetic, vascular, and epilepsies (Table 2).

## 4. Quantitative Analysis of Different Groups of the Cerebrospinal Fluid Metabolites Using GC–MS

As mentioned in the Introduction section, after metabolic profiling and various statistical and bioinformatics approaches, it is necessary to develop a selective method for the detection of a specific compound or group of related compounds. At this stage, modifications of sample preparation techniques are required to reduce the matrix effect and coextraction of nontargeted compounds.

Validation of the developed analytical methods for the quantitative evaluation of the potential biomarkers in a biological matrix is critical for the successful conduct of nonclinical and clinical studies, and it ensures that the obtained data are reliable. Validation includes the evaluation of a number of parameters, such as linearity, lower limit of detection and quantitation (LLOD and LLOQ), selectivity and specificity, sensitivity, accuracy, precision, recovery, and stability of the analyte in the matrix. However, full validation requires a large amount of the biological matrix. In the case of rare matrices, the validation can be performed on a pooled sample from several persons or a model solution that has a chemical composition similar to that of the matrix. We did not manage to find fully validated methods for the determination of the analytes in the CSF, which were performed according to the FDA or EMA guidelines. Several methods, such as recovery, LOQ and LOD values, and linearity will be discussed in this section (Table 3). However, most of the published methods do not provide enough validation data, and they will be mentioned in passing.

### 4.1. Amino Acids

The presence and levels of free amino acids in the CSF can be indicators of neurological diseases [4,47,64,77]. Silylation is commonly used for the amino acids and an alternative derivatization using a microwave-assisted derivatization was described [64]. A 200 µL aliquot of the artificial CSF (contains 127 µM NaCl, 2 µM KCl, 1.2 µM KH_2_PO_4_, 26 µM NaHCO_3_, 2 µM MgSO_4_, 2 µM CaCl_2_, 10 µM 4-(2-hydroxyethyl)-1-piperazineethanesulfonic acid (HEPES) and 10 mM glucose and bubbled with a carbogenic mixture (95% *v*/*v* O_2_ and 5% *v*/*v* CO_2_)) was used for the validation of this method for 16 amino acids (glycine, sarcosine, l-forms: alanine, valine, leucine, isoleucine, serine, threonine, methionine, aspartic acid, proline, cysteine, glutamic acid, phenylalanine, asparagine, and lysine) according to the Eurachem guidelines. Arginine and histidine were not analyzed because of the thermal instability of their derivatives. The evaluated analytical parameters, such as LOD 0.01–4.24 µM, LOQ 0.02–7.07 µM, intraday and interday precision values, recoveries, and linearity allowed the authors to determine all 16 amino acids in the human CSF samples (*n* = 16) at the level higher than LOD values, which indicated that the developed analytical method is applicable to solving the task of the quantitative determination of free amino acids in the CSF.

### 4.2. Tryptophan Metabolites

Tryptophan is one of the most important amino acid for CNS function. Its metabolism occurs in two main ways: the indole and kynurenine pathways. The indole metabolism is divided into the serotonin (5-hydroxytryptamine, 5-HT) via 5-hydroxytryptophan (5-HTP) and the microbial pathways. 5-Hydroxyindole-3-acetic acid (5-HIAA) or 5-hydroxyindole-3-ethanol (5-HTOL) appears as a result of the serotonin metabolism. The microbial pathway leads to the formation of metabolites containing an indole ring, for example, tryptamine and several indole-containing acids (indole-3-acetic (3IAA), indole-3-propanoic (3IPA), indole-3-carboxylic (3ICA), and indole-3-lactic (3ILA) acids).

5-HT is a neurotransmitter, and its related indole derivatives from serotonin pathway are involved in physiological and pathological responses, which are associated with many neurological diseases [46,58,59,60]. A method for detecting 5-HTOL, 5-HIAA, 5-HTP and 5-HT using solid-phase extraction (SPE) with Cleanert PEP-2 column was developed. This method required 3 mL of CSF, which seemed to be too much in the case of children, who were the participants of the study. However, many important analytical parameters (without reference to the FDA or EMA guidelines) were evaluated (matrix effect, linearity, LOD 0.1–0.4 µg/L and LOQ 0.5–2.0 µg/L, intraday and interday precision values, recovery, and coefficient of variation); these allowed the authors to obtain the statistically significant data about the changes in the concentration of the target compounds between children with acute lymphoblastic leukemia and the control group [46].

In contrast to the described method with classical SPE, a modern microextraction by packed sorbent (MEPS) with C18 was applied for the determination of the indole-containing acids (5-HIAA, 3IAA, 3IPA, 3ICA, and 3ILA) using only 40 μL of the CSF. The pooled CSF samples were used for the validation and the following parameters were evaluated (according to the FDA guidelines): linearity, recovery, LOD 0.2–0.4 µM and LOQ 0.4–0.5 µM, accuracy, precision, selectivity, and carryover effects. Despite the satisfactory results, only 3 IAA was detected in CSF samples of the patients with the CNS diseases [65].

Several studies describe an importance of the changes in the concentration of the kynurenine pathway metabolites, particularly pyridine-containing quinolinic, picolinic, and nicotinic acids, which are involved in the inflammatory and apoptotic processes associated with the CNS neuronal cell damage and death [50,51,52,53,66]. These metabolites were detected in the CSF after derivatization with trifluoroacetic anhydride and hexafluoroisopropanol, and electron-capture negative-ion chemical ionization (ECNI) GC–MS. One of these studies describes an almost fully validated method (LOQ less than 1 fmol for each of the analytes, linearity, precision, and accuracy) using for the concurrent quantification of quinolinic, picolinic, and nicotinic acids in 20–50 μL of model solutions and an artificial CSF [66].

### 4.3. Organic Acids

Phenyl-containing acids (benzoic, 3-phenylpropionic, 3-phenyllactic, 4-hydroxybenzoic, 2-(4-hydroxyphenyl)acetic, homovanillic, and 3-(4-hydroxyphenyl)lactic acids), which are mostly microbial metabolites of the tyrosine and phenylalanine, were detected in the CSF samples (*n* = 138) from neurosurgical patients (*n* = 84) with different CNS pathology using MEPS and traditional liquid–liquid extraction (LLE). The validation (linearity, recovery, LOD 0.1–0.3 µM and LOQ 0.4–0.7 µM, accuracy, precision, selectivity, and carryover effects) according to FDA guidelines was performed for both MEPS and LLE, demonstrating the equal possibilities of these sample preparation techniques. Similar results were achieved using 40 µL of the CSF sample for MEPS instead of 200 µL for LLE [67].

The detection of creatine, an N-containing acid, and its precursor guanidinoacetate is crucial in cases of creatine deficiency syndromes, a group of inherited metabolic disorders that are caused by abnormalities in creatine biosynthesis and/or transport [78]. A sample preparation technique included LLE from 100 μL of the CSF and derivatization with subsequent stable isotope dilution (SID) GC–MS. This method provides LOD and LOQ 0.0012 and 0.0024 μM for creatine, and 0.01 and 0.02 μM for guanidinoacetate, respectively. Linearity, interassay, and intra-assay variability were also evaluated. The reference values for creatine and guanidinoacetate were revealed and ranged from 17 to 78 μM and 0.02 to 0.56 for μM, respectively [63].

Gamma-hydroxybutyric acid (GHB) is a naturally occurring neurotransmitter and a precursor to gamma-aminobutyric acid (GABA), glutamate, and glycine in certain brain areas. The postmortem examination of the influence of temperature and time storage to in vivo production of GHB was evaluated using traditional LLE and silylation. The validation was performed using 50 μL of the CSF and resulted in LOD 0.5 mg/L, LOQ 0.6 mg/L, and interday and intraday accuracy ≥91%. GHB concentration changes were affected both during postmortem interval in the dead body and during in vitro storage [68].

GABA is the chief inhibitory neurotransmitter and plays an important role in various neurological and mental disorders, in which both elevated and decreased concentrations in CSF may occur. A sensitive, selective, and accurate SID GC-ECNI-MS method for the determination of free and total GABA was developed using 500 μL of the CSF, derivatization in aqueous solution with methylchloroformate, extraction with ethyl acetate, and derivatization of the dried residue with pentafluorobenzylbromide in acetonitrile and triethylamine (for free GABA). Total GABA determination included hydrolysis with sulphosalicylic acid during 24 h. The following analytical parameters were evaluated: LOD <0.005 µM and interassay and intra-assay variability for both free and total GABA. The applicability of the method was successfully demonstrated for the determination of free and total GABA in patients suffering from succinic semialdehyde dehydrogenase deficiency before and during specific treatment [69]. This method was used for the evaluation of free GABA in the CSF samples in a patient with pyruvate carboxylase deficiency [61] and for the determination of the pipecolic acid [70], a carboxylic acid of piperidine and one of the biomarkers of the pyridoxine dependent epilepsy [79].

Different polyunsaturated fatty acids are the components of neuronal and glial membrane phospholipids and participate in the development of Parkinson’s [45] and Alzheimer’s diseases [13]. Although the determination of these compounds includes traditional LLE and silylation, there are no validated methods for the CSF [13,45,62]. Furthermore, there are no validated methods for the determination of the nervonic acid, a candidate biomarker for depressive and manic symptoms [55], and *N*-acetylaspartic acid, a neuron-specific marker that is identified in multiple sclerosis [5].

### 4.4. Neuroactive Steroids

Neuroactive steroids are steroids synthesized de novo in the CNS and play a central role in neuronal processes [80]. Allopregnanolone and related neurosteroids (androsterone, dihydrotestosterone, testosterone, isopregnanolone, and pregnenolone) were detected as carboxymethoxime, pentafluorobenzyl, and trimethylsilyl derivatives using GC–ECNI-MS. The sample preparation included SPE with C18 sorbent from 1–2 mL of the CSF. Linearity, LOD 0.2–1.2 µg/L, recovery, and reproducibility were evaluated. This method was successfully applied for the analysis of the human and monkey CSF [56]. Another study was devoted to the evaluation of the correlations between peripheral and CSF steroids using a wide spectrum of bioactive steroids, their precursors and metabolites. Unconjugated steroids (pregnenolone, dehydroepiandrosterone, progesterone, androstenedione, testosterone, allopregnanolone, isopregnanolone, androsterone, epiandrosterone, 7α-hydroxy-dehydroepiandrosterone, 7β-Hydroxy-dehydroepiandrosterone, 5-androstene-3β, 7α, 17β-triol, 5-androstene-3β, 7β, 17β-triol, 16α-hydroxy-pregnenolone, 16α-hydroxy-dehydroepiandrosterone, and 16α-hydroxy-progesterone) were extracted from 1 mL of the CSF and derivatized in a common two-step procedure. LOD from 0.04 (for 5-androstene-3β, 7β, 17β-triol) to 11.3 pM (for androstenedione) were measured together with other analytical parameters. Significant correlations between some steroids in serum and CSF were revealed, particularly between the 7α/β-hydroxy-metabolites of dehydroepiandrosterone and androstenediol [72]. Another study applied similar sample preparation for the detection of free dehydroepiandrosterone and its 7-hydroxylated derivatives: 7α-hydroxy-dehydroepiandrosterone, 7β-hydroxy-dehydroepiandrosterone, 5-androstene-3β, 7α, 17β-triol, and 5-androstene-3β, 7β, 17β-triol [81]. Different neuroactive steroids were evaluated using GC–MS preceded by HPLC purification in Alzheimer’s disease [9], relapsing–remitting multiple sclerosis [42], and post-traumatic stress disorder [56].

### 4.5. Arachidonic Acid Metabolites

F_2_-isoprostanes (F_2_-IsoPs) and F4-neuroprotanes (F_4_-NPs) are compounds formed in vivo from the nonenzymatic free-radical-catalyzed peroxidation of essential fatty acids, primarily arachidonic and docosahexaenoic acids, respectively. Since CNS is characterized by a high level of polyunsaturated fatty acids and significant oxygen demand, considering its weak antioxidant defenses, it is also rather liable to oxidative damage caused by reactive oxygen or nitrogen species. Imbalance between free radicals and antioxidants, so-called “oxidative stress”, plays a crucial role in neurodegenerative disorders. F_2_-IsoPs and F_4_-NPs, being products of lipid peroxidation, can be biomarkers of oxidative stress and neurodegenerative diseases. The literature indicates that levels of F_2_-IsoPs and F_4_-NPs in CSF and brain tissue are elevated in case of such disorders as Alzheimer’s disease [11,14,82,83,84] and equine neuroaxonal dystrophy [85]. For sensitive quantification of these compounds, which are present in the CSF samples in low concentrations, GC–MS methods are applied [86], and their validation is required not only for urine and serum [87], but for CSF as well.

Prostanoids, which include prostaglandins and thromboxanes, are the metabolites of the enzymatic pathways of arachidonic acid. These compounds have similar chemical structures but different biological and therapeutic effects. Simultaneous assay of these compounds in the CSF was developed, which included extraction with octadecyl silica gel and two-step purification with silicic acid gel chromatography [48]. Some representatives of this class of compounds were elevated in patients with multiple sclerosis [6].

The validated analytical procedures for different metabolites of arachidonic acid are required because of the high demand in their evaluation in different neurodegenerative disorders.

### 4.6. Glucose Metabolites

Abnormalities in carbohydrate metabolism are of interest in mood disorders studies, as a possible relationship between diabetes and major depression has been shown. Glucose, which serves as an energy source for cells, is conversed to fructose via the polyol pathway with sorbitol being an intermediate compound formed during this two-step process. The CSF sorbitol levels were investigated in patients with bipolar and unipolar mood disorder, and sorbitol concentrations were higher in the CSF of depressed subjects compared to normal controls [54]. Sorbitol levels along with fructose levels, both being glucose metabolites, were found to be elevated in the CSF of multiple sclerosis patients as well, while concentrations of myoinositol that is not produced via the polyol pathway did not differ significantly from its concentrations in the CSF of control subjects [3]. No validation data were demonstrated; thus, the development of the validated analytical method for the determination of the glucose metabolites is required.

### 4.7. Drugs and Toxic Metabolites

Indomethacin is a nonsteroidal anti-inflammatory drug, mainly known for its ability to inhibit cyclooxygenase, which is responsible for the prostaglandins production catalysis. Indomethacin is often prescribed to treat inflammation and pain caused by rheumatic and orthopedic diseases or surgery. To determine its concentrations in 250 μL of CSF, a SPE sample preparation technique was applied followed by derivatization with pentafluorobenzylbromide and GC–NICI–MS. The method provides moderate analytical characteristics (recoveries, accuracy, intraday precision) with LOQ 0.1 ng/sample. The CSF indomethacin levels in healthy children were found to be 0.2 and 5.0 ng/mL after administering it intravenously [73].

GC–MS was also used for determination of another drug: ELND005 (scyllo-inositol), an endogenous inositol stereoisomer. This drug could be used for Alzheimer’ disease treatment, and its pharmacokinetic behavior in the CSF after oral administration was of interest. A traditional combination of protein precipitation, LLE, and derivatization was used for sample treatment, and validation resulted in LLOQ 0.4 μg/mL (linearity, precision, and accuracy were also evaluated) [74].

Analysis of the CSF could also be beneficial for toxicology studies. For instance, morphine can be found in human biological samples and tissues due to the ingestion of heroin or codeine, since these compounds are both metabolized to morphine, or because of exposure to morphine itself. To distinguish whether it was heroine or morphine administering, determination of 6-monoacetylmorphine is often used. However, it converses to morphine rather rapidly, and its concentrations in blood may be lower than the LOD of the method used for 6-monoacetylmorphine determination. Several studies suggested that 6-monoacetylmorphine persists in the CSF and some other human biological samples when compared to blood. 6-Monoacetylmorphine, free morphine, and free codeine levels were investigated in the CSF samples in 25 heroin deaths. The sample pretreatment procedure included such steps as SPE and derivatization and was combined with GC–MS analysis. The method’s LOQ for 6-monoacetylmorphine was 0.001 mg/L; linearity and precision were evaluated. 6-Monoacetylmorphine levels were 6.6 times higher on average in the CSF samples than in blood [75].

Analysis of the diethylene glycol and its potential metabolites (ethylene glycol, glycolic acid, oxalic acid, diglycolic acid, and hydroxyethoxy acetic acid) is required because of the human poisoning during misformulation into pharmaceutical products. Sample preparation for acid metabolites from 100 μL of the CSF included traditional LLE and silylation. Sample preparation for diethylene and ethylene glycol from 250 μL of the CSF included extraction and derivatization with pentafluorobenzoyl chloride with the following analysis by GC–NICI–MS. The LOQ values were 0.05–1.0 µg/mL; accuracy and linearity were evaluated [76].

Different low-molecular-weight compounds are required to be detected in the CSF for the diagnosis of the CNS diseases. The literature data analysis revealed the absence of fully validated methods, and it presents opportunities for scientists to develop and validate analytical protocols using modern sample preparation techniques.

## 5. Miniaturization in Sample Preparation Techniques for the GC–MS Analysis

A small sample volume is one of the main criteria for the analysis of CSF. There are interesting approaches to sample preparation of biological fluids for GC–MS analysis, which use the principle of miniaturization.

The method of homogenous liquid–liquid microextraction (HLLME), compared to the classical LLE using significant volumes of organic solvents, is based on the extraction of polar organic compounds from aqueous matrices, including biofluids, with small volumes (microliters) of water-miscible organic solvents. For the phase separation, the salting-out effect is often used, followed by centrifugation. The volumes of biological fluids are usually up to hundreds of microliters and the volumes of polar solvents are often several times less; the weighed portion of the salting-out agent is tenths of a gram. The achievable LOD of analytes are nanograms and tenths of nanograms per milliliter [88]. The method could be combined with HPLC–MS [89], but a combination with GC–MS is also possible. To determine volatile analytes without derivatization, it is necessary to dry the extract by adding anhydrous sodium sulfate. Analytes can be derivatized both directly in the extract (addition of chloroformates) and in the dried extract (silylation in acetonitrile).

Dispersive liquid–liquid microextraction (DLLME) is based on the extraction of analytes with a microemulsion followed by the phase separation. Usually, the volume of a mixture of an extracting solvent and a dispersing solvent is hundreds of times less than the volume of the analyzed solution. Analytes are extracted quantitatively at high preconcentration factors. Extraction equilibrium is established in minutes [90]. Various methods of dispersion have been proposed and various extracting solvents have been studied for a large number of different matrices, including biological ones [91,92]. The DLLME method is applicable for the analysis of small volumes of analyzed solutions and could be combined with derivatization procedure for polar and/or nonvolatile analytes with small volumes of reagents. Different derivatizing agents are used, e.g., for analysis of human urine, ethyl chloroformate in pyridine was utilized to convert 20 amino acids into their volatile carbamate esters, which were further analyzed using GC–MS. The derivatization process was carried out simultaneously with DLLME using trichloroethylene and acetonitrile as extracting and dispersing solvent, respectively. The range of LOD was 0.4–3.7 μg/L [93].

An interesting solution is to combine the capabilities of DLLME and injector port silylation technique for the determination of polar analytes in biological matrices. Aliquots of the extract and derivatizing reagent are injected simultaneously or sequentially into a heated GC–MS injector. A gas phase reaction occurs between the silylating reagent and polar analytes at the injector temperature. This approach reduces the derivatization time (less than a minute), the possibility of derivatives decomposition, and the amount of toxic reagent and solvents used for the process is smaller [94].

QuEChERS (the name is formed from “quick, easy, cheap, effective, rugged, and safe”) is a two-step process involving liquid extraction (usually using acetonitrile) and dispersive SPE (dSPE) using (more commonly) primary secondary amines, C_18_, and/or graphitized carbon black sorbents to eliminate significantly interfering matrix components (for example, humic acids, lipids, etc.). QuEChERS, originally developed for the extraction of acidic and basic pesticides from food [95], is also used for analysis of blood plasma samples. A method for the simultaneous extraction of acidic, basic, neutral, and amphiphilic analytes from blood plasma using a micro version of QuEChERS (micro-QuEChERS) is proposed. It reduces the volumes of samples (200 μL of plasma compared to 1.5 mL required in the nonmicro version) and reagents by 8 times. The method allowed for the extraction of analytes from blood plasma with high (65 to 80%) recoveries and low matrix effect. The developed approach is considered to be a fast and clean alternative to “dilute and shoot” approaches or the protein precipitation procedure, which are used for high-throughput clinical diagnostics (including analysis of the CSF samples), coupled with HPLC–MS. However, it is of interest to study a possible combination of micro-QuEChERS with GC–MS [96].

Micro-QuEChERS was used to analyze 148 avian blood samples collected in an environmental field study of the impact of rodenticides (applied for the treatment of common vole plague) on the wildlife. The volume of each sample was 250 μL. In combination with GC–MS/MS, this method detected the desired analytes at the level of 1.5 ng/mL [97]. Although micro-QuEChERS is not yet widely used for sample preparation of biological matrices [95,96,97,98], the ability to vary components for the extraction and extracts purification procedures applying dSPE, reduce the cost and time of analysis, and analyze relatively small sample volumes (even smaller than those described in the publications above) presents broad prospects for the analysis of biological fluids, including CSF [96,97,98].

Solid-phase microextraction (SPME) is a solvent-free sample preparation technique [99]. This method is widely used for analyzing the vapor phase of various biological fluids in order to determine volatile organic compounds and to extract analytes directly from the liquid phase [100,101,102]. Substances are absorbed by a polymer film or solid sorbent covering a piece of fiber (a piece of fused silica capillary). The capillary is placed inside a needle connected to a syringe-like device. During sorption and desorption, the capillary moves out of the needle. The metabolomic composition of the circulating blood of laboratory mice was investigated [100], and for selective recovery of hydrophilic and hydrophobic analytes with respect to high molecular weight matrix components, an SPME fiber coated with mixed-mode polymers (phenylboronic acid and polystyrenedivinylbenzene) was used. The fiber was placed in an injection needle, and it absorbed the metabolites directly from the bloodstream. The vapor phase over saliva samples (sample volume 500 μL) was investigated. The LOD for the 20 detected volatile metabolites ranged from 0.008 to 1 µM [101]. The vapor phase over urine samples was studied in order to identify biomarkers of cancer; 82 metabolites were found, and the sample volume was 4 mL [102]. The SPME capabilities allow the researchers to vary the volumes of the studied samples, depending on the aims of the research.

Stir bar sorptive extraction was also developed as another solvent-free sample pretreatment technique, and it is actively used for isolation of low-molecular-weight components of different polarity from biological fluids [103,104,105]. The device used for stir bar sorptive extraction is a glass tube with a metal rod inside (magnetic stirrer), often coated with polydimethylsiloxane. The main difference between stir bar sorptive extraction and SPME is the larger amount of stationary phase covering the surface of the mixer (up to 25 μL) compared to the capillary cut (0.5 μL), which increases the extraction efficiency. Desorption of analytes is carried out either by solvent re-extraction or thermal desorption. For polar components in biological matrices, it is possible to combine simultaneous deconjugation and extraction of analytes (in situ deconjugation) or derivatization and extraction (in situ derivatization) followed by thermal desorption into the GC injector. Derivatization could also be carried out after extraction (postextraction derivatization), both during thermal desorption of analytes and after their re-extraction. Sample volumes are typically on the order of 1 mL, with LOD being attainable from ng/mL to pg/mL.

A method of amino acids microextraction from biological fluids (including CSF), based on a combination of hollow fiber SPME and extraction with stir bar sorptive extraction—hollow fiber–stir bar sorptive extraction—was proposed [106]. Hollow polymer fibers are obtained using the coaxial electrospinning technology when an electrostatic field acts on an electrically charged jet of a polymer solution or its melt. It is also possible to obtain a hollow fiber membrane with specified properties (average pore diameter, membrane thickness), providing a semipermeable barrier (analytes pass through the pores of the membrane, the matrix components remain in solution). For the first time, the hollow fiber membrane was used as a SPME fiber coating for the extraction of BTEX (benzene, toluene, ethylbenzene, and xylenes) from aqueous matrices, and was based on a polypropylene coated copper wire [107].

To extract amino acids, hollow polyvinylidene fluoride fiber was used. A piece of polyvinylidene fluoride hollow fiber was sealed on the one side, and a steel rod was placed inside the fiber. A dispersion system of 0.1 g of silica microspheres in ethanol was introduced into the fiber. After removal (including evaporation) of ethanol, the fiber was sealed on the other side. Before use, the resulting hollow fiber–stir bar sorptive extraction device was washed with acetone and dried. An aliquot of the biological fluid was mixed with ethanol in a 3:1 ratio in order to reduce the surface tension of the sample to facilitate the penetration of analytes into the membrane micropores. A hollow fiber–stir bar was placed in a vial with the sample and extraction was performed while stirring under the influence of a magnetic field for a chosen time. During this time, amino acids selectively with respect to biological macromolecules penetrated from the solution of biological fluid through the pores of the membrane and were absorbed on the surface of silica microspheres due to the formation of hydrogen bonds. Next, the hollow fiber–stir bar was removed from the sample vial, dried until moisture was completely evaporated, placed in another vial, and 0.1 mL of BSTFA and 0.9 μL of a nonpolar solvent were added to extract amino acid derivatives, which should have been formed as a result of silylation. BSTFA molecules also penetrated the pores of the membrane and interacted with amino acid molecules. Derivatization was carried out in a microwave field. The resulting derivatives were extracted with a nonpolar solvent and analyzed using GC–MS. Before the next extraction, the hollow fiber–stir bar was conditioned in distilled water and acetone. The LOD of the studied amino acids ranged from 3 × 10^−4^ to 6 × 10^−3^ µg/mL. Recoveries from the rat CSF samples ranged from 71.8 to 101.2%. The resulting extraction device could be used 30 times without loss of analyte sensitivity [106]. Due to the miniaturization of the device, it can be used for small volumes of biological fluids. One of the advantages of this method is that there is no need in sample cleanup.

One of the perspective sample preparation techniques is a MEPS [108] that is also aimed at miniaturizing SPE. The method is based on the use of a small amount of sorbent (1–4 mg) and multiple passage of the test sample through this sorbent layer located in the extension of the syringe needle. The method requires small amounts of sample (tens, hundreds of microliters), and solvent volumes (up to tens of microliters) which eluate analytes. This technique minimizes the dead volume as well. The syringe is placed in an automatic dispenser, and the speed and number of cycles of passing the sample through the sorbent could be programmed. It is also possible to connect the dispenser to an HPLC or GC system. A large number of needles with sorbents, which are common for classical SPE (C_18_, C_8_, silica gel), have been developed and are commercially available. A method for the determination of low-molecular-weight metabolites using a hand-operated device with hypercrosslinked polystyrene was described [109]. MEPS has found wide application in analytical chemistry, including the field of biological fluids analysis [110,111,112,113]—in particular, for the determination of low-molecular-weight microbial metabolites in the CSF (see Section 4) [65,67].

The CSF samples are characterized not only by low levels of the analytes of interest, but also by small sample volumes available for analysis. Miniaturization of classic extraction methods, such as LLE and SPE which are often used for analysis of biological fluids, coupled with GC–MS presents new perspectives in the metabolic analysis of the CSF.

## 6. Conclusions

GC–MS plays an important role in the development of the metabolic analysis of biological fluid samples. High separation efficiency and detection sensitivity, stable retention times, and reproducible mass spectra of analytes make it possible to analyze multicomponent mixtures of low-molecular-weight organic compounds of complex composition and to perform nontargeted and targeted analysis. Sample preparation based on the selective extraction of analytes with respect to interfering matrix components is often required to detect a wide range of components by this method. The complexity of the CSF analysis is caused not only by the low content of the target analytes, but also by the small volume of samples available for analysis. The miniaturization of the traditional LLE and SPE methods in combination with various options for the derivatization of polar analytes presents new possibilities in the metabolic analysis of the CSF using GC–MS.

## Figures and Tables

**Table 1:** The CSF metabolic profiling using GC–MS methods (CSF volume, samples preparation, and type of capillary column).

Aim	GC–MS Method, Capillary Column	CSF Sampling	Compounds	Sample Volume	Sample Preparation	Reference
Presenting a catalog of detectable metabolites (including their concentrations and disease associations) that can be found in human CSF. This catalog was assembled using a combination of both experimental (NMR, GC–MS, LC–FTMS) and literature-based research.	GC–MS: DB-5 column	Patients screened for meningitis (*n* = 50).	41 metabolites: amino acids, fatty acids, steroids, carbohydrates, et al.	200 µL	CSF + 800 µL 8:1 HPLC-grade MeOH-deionized water + vortexing + centrifugation + 200 µL of the supernatant was evaporated to dryness + 40 µL methoxyamine hydrochloride + incubation 90 min at 30 °C + 40 µL MSTFA + 20 µL proline IS + incubation at 30 °C for 45 min.	[22]
Update of the CSF metabolome database. Determination of metabolites using different methods, including NMR, GC–MS, LC–FTMS, direct flow injection–MS/MS and ICP–MS.	GC–MS: DB-5 column	Patients screened for meningitis (*n* = 7).	The same as in [22]	200 µL	The same as in [22].	[30]
Analysis of protein and metabolite abundances in CSF by multiple analytical platforms. Integration of metabolomics and proteomics to present biological variations in metabolite and protein abundances and compare these with technical variations with the currently used analytical methods.	GC–MS: 30 m × 0.25 mm × 0.25 μm, HP5-MS	Subjects (*n* = 9), the validation sample set (*n* = 28), and the experimental sample sets (*n* = 36 for proteomics and *n* = 42 for metabolomics).	93 metabolites: amino acids, organic acids, nucleosides, fatty acids, mono- and disaccharides, et al.	60–100 μL	60 μL CSF + 250 μL MeOH + centrifugation. 100 μL CSF samples from the validation sample set + 400 μL MeOH + drying under N_2_ + derivatization with MSTFA in pyridine. The final volume was 45 μL for the original sample set and 135 μL for the validation sample set.	[31]
To conduct a global metabolomics analysis to provide an overview of the postprandial alterations in CSF and plasma metabolites and to facilitate the application of CSF for biomarker screening (using metabolomics).	GC–MS/MS: 30 m × 0.25 mm × 0.25 μm, DB-5MS-DG.	Healthy subjects (*n* = 9). CSF collected both preprandial and postprandial CSF. The postprandial time was set at 1.5 h (*n* = 3), 3 h (*n* = 3), and 6 h (*n* = 3).	150 metabolites: amino acids, fatty acids, indoles, carbohydrates, et al.	500 µL	CSF + 9 volumes MeOH + centrifugation + IS + drying under N_2_ + oximation and trimethylsilylation.	[32]
Study on the effects of preanalytical factors on the porcine CSF proteome and metabolome using a variety of techniques comprising LC–MS, GC–MS, and MALDI-FT-ICP-MS.	GC–MS: 30 mm × 0.25 mm × 0.25 μm, HP5-MS.	Conventional pigs (*n* = 5).	49 metabolites: amino acids, sugars, hydroxy acids, et al.	100 µL	Lyophilization + derivatization with ethoxyamine hydrochloride in pyridine + derivatization with MSTFA.	[33]
Characterization of the metabolites present in CSF and comparison of metabolite levels in patient-matched setting to those found in serum.	GC × GC–TOFMS: 10 m × 0.18 mm × 0.18 µm, Rxi-5ms; 1.5 m × 0.1 mm × 0.1 µm. BPX-50.	Healthy subjects (*n* = 53).	1280 metabolites, quantitatively determined 21 compounds	25 µL	25 µL CSF + 10 µL IS + 400 µL MeOH + vortexing + centrifugation + 30 min at –20 °C + drying under N_2_ + derivatization 25 µL *O*-methylhydroxylamine hydrochloride + incubation 60 min, 45 °C + 25 µL MSTFA + incubation 60 min at 45 °C + hexane.	[34]
A GC–MS-based metabolomic analysis of CSF samples from glioma patients.	GC–MS: 30 m × 0.25 mm × 1.0 μm, DB-5, 30 m × 0.25 mm × 0.25 μm, CP-SIL 8 CB low bleed/MS.	Patients with intracranial glial tumors (*n* = 32).	45 metabolites, quantitatively determined 16 compounds.	50 µL	50 µL CSF + 250 µL MeOH–water–chloroform (2.5:1:1) + IS + vortexing + centrifugation + 250 µL of supernatant + 200 µL distilled water + vortexing + centrifugation + 250 µL of supernatant was lyophilized + 40 µL 20 mg/mL methoxyamine hydrochloride in pyridine + 20 µL MSTFA + centrifugation.	[23]
Exploring potential biomarkers and improving understanding of biochemical features of CSF-mediated autoimmune inflammatory diseases of the CNS.	GC–TOFMS: RTX-5Sil MS.	Patients suspected to have inflammatory demyelinating diseases (*n* = 145), control subjects without medical or neurological illness (*n* = 12)	962 metabolic signatures, quantitatively determined 85: sugars and sugar alcohols (24%), amino acids (28%), fatty acids (15%), organic acids (15%), amines (2%) et al.	100 μL	100 μL CSF on ice at 4 °C + 650 μL MeOH–isopropanol–water, 3:3:2, *v*/*v*/*v* + centrifugation + 700 μL of supernatant + drying + storage at –80 °C until derivatization + 5 μL 40 mg/mL methoxyamine hydrochloride in pyridine + incubation 90 min at 200 rpm, 30 °C + 2 μL IS + 45 μL MSTFA+1% TMCS + incubation 1 h at 200 rpm, 37 °C.	[26]
CSF metabolome study in a group of patients with different clinical and genetic subtypes of amyotrophic lateral sclerosis using GC–TOFMS.	GC–TOFMS: 10 m × 0.18 mm × 0.18 μm DB 5-MS.	amyotrophic lateral sclerosis patients (*n* = 78), healthy subjects (for control)	120 peaks, 40 identified.	100 μL	100 μL CSF + 900 μL IS and MeOH–water, 1:9 + 11 IS + beadmill for 1 min (90 Hz), 2 h on ice + centrifugation + 200 μL evaporized + storage at –80 °C + 30 μL (15 μg/μL) methylhydroxylamine hydrochloride 98% in pyridine + vortexing + heating 70 °C, 1 h + 16 h at room temperature + 30 μL MSTFA+1% TMCS, room temperature, 1 h + 30 μL heptane.	[25]
A detailed analytical evaluation of GC–APCI–TOFMS. In addition to the detailed examination of the analytical performance (repeatability, reproducibility, linearity, and detection limits), the applicability of this technique for metabolic profiling of CSF was demonstrated.	GC–APCI–TOFMS 30 m × 0.25 mm × 0.25 μm HP-5-MS.	Human CSF	300 compounds, 21 identified	250 μL	250 μL CSF + 600 μL MeOH + centrifugation + evaporation + 100 μL methoxyamine:pyridine mixture, 40 °C, 60 min +100 μL BSTFA or MSTFA containing 1% TMCS, 40 °C, 30 min + 2 h equilibration.	[35]
GC–MS/MS-based metabolome analysis of the CSF in pediatric patients with and without epilepsy.	GC–MS/MS	Patients with epilepsy (*n* = 34), patients without epilepsy (*n* = 30)	180 metabolites	50 μL (from reference)	(From reference): 50 µL serum + 250 µL MeOH–water–chloroform (2.5:1:1) + shaking, 30 min at 37 °C + centrifugation + 225 µL supernatant + 200 µL distilled water + centrifugation + 250 µL + 40 µL 20 mg/mL methoxyamine hydrochloride in pyridine + shaking + 20 µL MSTFA + incubation 30 min, 1200 rpm at 37 °C + centrifugation.	[36]
Testing the hypothesis that fatty acid metabolism in Alzheimer’s disease or mild cognitive impairment is altered compared to cognitively healthy study participants, and that details of the changes could be revealed by study of the brain-derived nanoparticles and supernatant fluid fractions of CSF.	GC–MS: 30 m × 0.25 mm × 0.50 μm, Phenomenex Zebron ZB-1MS.	Total participants (*n* = 139): cognitively healthy (*n* = 70), mild cognitive impairment (*n* = 40), Alzheimer’s disease (*n* = 29).	20 fatty acids (6 saturated, 6 monounsaturated, 8 polyunsaturated fatty acids)	1 mL	1 mL CSF + 100 ng IS + formic acid (0.9%, 3 drops) + lipid extraction + 0.5 mL chloroform:MeOH solution (1:1, *v*/*v*), 0.5 mg/mL butylated hydroxytoluene + vortexing + storage –40 °C + PFBBr in MeCN solution (1:19 *v*/*v*, 50 μL) and diisopropyl ether in MeCN solution (1:9 *v*/*v*, 50 μL), 20 min at 45 °C + drying under N_2_ + 1 mL hexane.	[10]
Investigation of CSF and plasma metabolomic profiles for prediction of Parkinson’s disease progression.	GC–MS	Participants with relatively mild Parkinsonism. Donors (*n* = 49) were randomly selected placebo-treated participants.	383 biochemicals	No data	No exact information, except references to the earlier publications. Brief information describes extraction and derivatization with BSTFA.	[8]
Investigation of the metabolomics profile of patients affected by relapsing–remitting multiple sclerosis and primary progressive multiple sclerosis, in order to find potential biomarkers to distinguish between the two forms.	GC–MS: 30 m × 0.25 mm × 0.25 μm, TG-5MS.	Patients (relapsing–remitting multiple sclerosis *n* = 22, primary progressive multiple sclerosis *n* = 12)	Different classes of compounds (data most discussed in this article were obtained from LC–MS and flow injection–MS analysis).	200 μL	200 μL + lyophilization + drying + 50 μL methoxyamine in pyridine (10 mg/mL), 70 °C + 100 μL MSTFA, room temperature, 1 h + 100 μL hexane.	[24]
Comprehensive analysis of the absorbed constituents in the plasma and CSF of rabbits after intranasal administration of Asari Radix et Rhizoma by headspace solid-phase microextraction–GC–MS and HPLC–atmospheric pressure chemical ionization–ion trap-time of flight-multistage mass spectrometry (HPLC–APCI–ion-trap-TOF-MSn).	GC–MS: 30 m × 0.25 mm × 0.25 µm, Rxi-5MS.	Rabbits (*n* = 15)	25 metabolites	500 µL	500 µL CSF in 10 mL headspace vial + 0.10 g NaCl + polydimethylsiloxane/divinylbenzene fiber was exposed to the headspace at 70 °C, 40 min + fiber was withdrawn into the needle + desorption at 250 °C for 3 min into the GC injection port.	[37]
Investigation of CSF metabolomics in an acute experimental autoimmune encephalomyelitis rat model using targeted LC–MS and GC–MS.	GC–MS: 30 m × 0.25 mm × 0.25 μm, HP5-MS.	Rats (*n* = 84)	14 amino acids and related compounds	30 μL	30 μL CSF + 250 μL MeOH + centrifugation + drying under N_2_ + derivatization with MSTFA in pyridine. The end volume was 45 μL.	[2]
A nontargeted metabolomic analysis using GC–MS was conducted to identify differentially expressed metabolites between naturally occurring depressive and control macaques.	GC–MS: 30 m × 0.25 mm × 0.25 μm, HP-5MS.	Naturally occurring depressive female macaques (*n* = 10) and age- and gender-matched healthy controls (*n* = 12)	663 variables, 37 metabolites	15 μL	~15 μL CSF + 10 μL IS + vortexing + 90 μL MeOH + centrifugation + 95 μL of supernatant + drying under N_2_ + 30 μL methoxamine hydrochloride (20 mg/mL pyridine) + incubation 37 °C, 90 min + 30 μL of BSTFA + 1% TMCS 70 °C, 60 min + cooling to room temperature.	[38]
Report on an analytical method that can be used for metabolomics studies when only a limited amount of sample volume is available.	GC–MS: 30 m × 0.25 mm × 0.25 μm, HP-5MS.	Rats (*n* = 60, total number of CSF samples *n* = 90)	93 metabolites, 73 identified: fatty acids, amino acids, tricarboxylic cycle acids, carbohydrates, polyols, purine/pyrimidine bases, et al.	10 μL	10 μL CSF + 40 μL MeOH + centrifugation 10 min, 11,800 rpm + drying under N_2_ + 10 μL ethoxyamine·HCl (*c* = 56 mg/mL (0.58M) in pyridine), 90 min, 40 °C + 20 μL MSTFA, 50 min, 40 °C. The final volume was 50 μL.	[39]
Presentation of an analytical method using in-liner silylation coupled to GC–MS that is suitable for metabolic profiling in ultrasmall sample volumes of 2 μL down to 10 nL.	GC–TOFMS: 30 m × 0.25 mm × 0.25 μm HP5-MS.	Mouse and human CSF samples.	342 peaks, 52 identified in human CSF: amino acids, organic acids, fatty acids, sugars, et al.	2 μL	The microvials containing the dried sample were placed inside the PTV injection liner + 1 μL IS + 3 μL MSTFA.	[40]
Application of untargeted metabolomics using GC–TOFMS to the CSF of aneurysmal subarachnoid hemorrhage patients to determine global metabolic changes and metabolite predictors of long-term outcome that are independent of vasospasm status.	GC–MS: 30 m × 0.25 mm × 0.25 μm, Rtx-5Sil MS.	Patients with aneurysmal subarachnoid hemorrhage (*n* = 15).	97 metabolites	5 µL	5 μL of CSF + 1.0 mL MeCN, isopropanol, and water in proportion 3:3:2 + vortexing + centrifugation + drying + 450 μL degassed 50% MeCN + centrifugation + derivatization.	[41]

**Table 2:** Candidate low-molecular-weight biomarkers, discovered using GC–MS methods, for different types of diseases.

Disease Classification	Diagnosis	Candidate Biomarkers	Reference
Inflammatory demyelinating	Multiple sclerosis	5 amino acids, *O*-phosphoethanolamin	[2]
		sorbitol, fructose	[3]
		homocysteine	[4]
		*N*-acetylaspartic acid	[5]
		2 metabolites of arachidonic acid	[6]
		quinolinic acid, picolinic acid	[7]
		3 neuroactive steroids	[42]
		4 endocannabinoids	[43]
	Neuromyelitis optica spectrum disorder and idiopathic transverse myelitis data	2 monoglycerides, salicylaldehyde, 4 organic acids, inosine, threose, butane-2,3-diol, hypoxanthine, glutamine	[26]
Neurodegenerative	Amyotrophic lateral sclerosis	amino acids, organic acids	[25]
	Alzheimer’s disease	2 steroids	[9]
		fatty acids	[10]
		8,12-iso-iPF2α-VI	[11]
		total isoprostane iPF2α-VI	[12]
		polyunsaturated fatty acids	[13]
		F2-IsoPs	[14]
	Multiple system atrophy	7 polyamines	[44]
		eicosapentaenoic acid	[45]
Oncological	Glioma	citric and iso-citric acid	[23]
	Leukemia	5-hydroxytryptamine, 5-hydroxyindole acetic acid	[46]
Infectious	Meningitis	5 amino acids	[47]
		prostaglandins, thromboxane B_2_	[48]
		muramic acid	[49]
		quinolinic acid, picolinic acid	[50]
	Malaria		
			[51]
	HIV-associated impaired prospective memory	quinolinic acid	[52]
	Subacute sclerosing panencephalitis	quinolinic acid	[53]
Mental	Mood disorders	sorbitol	[54]
		fatty acids, amino acids	[38]
	Major depressive disorder	nervonic acid	[55]
	Post-traumatic stress disorder	allopregnanolone, pregnanolone	[56]
		allopregnanolone, pregnanolone	[57]
	Diagnosis of suicidal behavior	5-hydroxyindolacetic acid	[58]
		homovanillic acid	[59]
		5-hydroxyindolacetic acid, homovanillic acid	[60]
Genetic	Pyruvate carboxylase deficiency	free-gamma-aminobutyric acid, glutamine, C5 ketone bodies	[61]
	Combined sepiapterin reductase and methylmalonyl-CoA epimerase deficiency	2 polyunsaturated fatty acids	[62]
	Guanidinoacetate methyltransferase (GAMT) and creatine transporter deficiency	guanidinoacetate	[63]
Vascular	Aneurysmal subarachnoid hemorrhage	free amino acids	[41]
Epilepsies	Epilepsy	2-ketoglutaric acid, pyridoxamine, tyrosine, 1,5-anhydro-glucitol	[36]

**Table 3:** The analytical methods for the determination of the low-molecular-weight compounds in the CSF using GC–MS methods.

Compounds	GC–MS Method, Capillary Column	CSF Sampling	Sample Volume	Sample Preparation	Method Validation	Concentration	Reference
Glycine, sarcosine, l-forms: alanine, valine, leucine, isoleucine, serine, threonine, methionine, aspartic acid, proline, cysteine, glutamic acid, phenylalanine, asparagine, lysine.	GC–MS: 30 m × 0.25 mm × 0.25 µm Rtx-5MS.	Artificial CSF, patients (*n* = 16).	200 µL	200 µL CSF + 800 µL MeOH at –10 °C + vortexing + centrifugation + 200 µL supernatant + evaporation to dryness at room temperature under N_2_ + 15 µL methoxyamine in pyridine (20 mg/mL) + 35 µL BSTFA+TMCS (99:1 *v*/*v*) + vortexing + derivatization under microwave irradiation, 210 W, 3 min.	Recovery: 88–129%. LOD: 0.01–4.24 µM. LOQ: 0.02–7.07 µM. Intraday (RSD): 4.1–15.6%. Interday (RSD): 6.4–18.7%. Lin. 0.1–133.0 µM (*R*^2^ = 0.99 for amino acids except cysteine).	Median (*n* = 16), µM: 6.9/4.9/19.9/9.3/6.7/4.2/7.9/7.6/10.4/6.2/375.0/1046.7/13.0/4.8/1.1/10.4.	[64]
5-hydroxyindole ethanol (5-HTOL), 5-hydroxyindole acetic acid (5-HIAA), 5-hydroxytryptophan (5-HTP), 5-hydroxytryptamine (5-HT).	GC–MS: 30 m × 0.25 mm × 0.25 μm, DB-5.	Children with acute lymphoblastic leukemia without chemotherapy (*n* = 36), control group (*n* = 24)	3 mL	3 mL CSF + SPE + washing + elution 1 mL MeOH + 0.5% formic acid + drying under N_2_ + 70 μL BSTFA+1% TMCS + 30 μL pyridine + 2.5 μL MeOH + incubating for 1 h at 95 °C.	Matrix effect: 92.3–106.2% (no significant ME). Lin. 0.5–200.0 µg/L (5-HTOL, 5-HIAA, *R*^2^ ≥ 0.9924) and 2.0–800.0 µg/L (5-HTP, 5-HT, *R*^2^ ≥ 0.9918). LOD: 0.1–0.4 µg/L. LOQ: 0.5 (5-HTOL, 5-HIAA) and 2.0 (5-HTP, 5-HT) µg/L. Intraday recovery: 94.6–105.6% (CV 1.4–4.5%). Interday recovery: 93.0–106.9% (CV 1.8–4.5%).	Children with acute lymphoblastic leukemia without chemotherapy4.3/61.0/5.3/3. Control group: 4.5/88.9/5.8/6.5	[46]
Indole-3-carboxylic (3ICA), indole-3-acetic (3IAA), indole-3-propionic (3IPA), indole-3-lactic (3ILA), 5-hydroxyindole-3-acetic (5-HIAA) acids.	GC–MS: 30 m × 0.25 mm × 0.25 µm, TR-5ms.	CSF (*n* = 3) samples of different patients with CNS diseases.	40 μL	40 µL CSF + 40 µL distilled water + MEPS + elution with diethyl ether + drying + 40 μL BSTFA/MTBSTFA + incubation 30 min at 90 °С + cooling 30 min at 4 °С + 350 μL of hexane.	Recovery: 40–80% (for pooled CSF). LOD: 0.2–0.4 µM. LOQ: 0.4–0.5 µM. Precision (RSD): <20%. Accuracy (the relative error, RE): <±20% (at the LOQ concentrations). Lin.: 0.4–7 µM (*R*^2^ ≥ 0.9949).	3IAA, µM: 0.42 ± 0.08; 0.6 ± 0.1; 0.43 ± 0.03	[65]
Quinolinic, picolinic, nicotinic acids.	GC–ECNI-MS: 30 m × 0.25 mm, HP–5MS (i) 0.25 μm or (ii) 1.0 μm stationary-phase film thickness.	Human CSF samples, artificial CSF	20–50 μL	CSF + evaporation to dryness + 100 μL trifluoroacetic anhydride + 100 μL hexafluoroisopropanol + heating at 60 °C for 30 min + dissolving in 1 mL toluene + washing with 1 mL 5% NaHCO_3_ + 1 mL water + ~500 mg anhydrous Na_2_SO_4_.	On-column LOQ: < 1 fmol (S/N 10:1). Lin.: 0–5 pmol on column. Slope: for nicotinic acid 5.8; for picolinicacid 25.8 (*R*^2^ > 0.996). Precision (RSD): 0.5–4.3%. Accuracy: 94.0–105.5%. Interday precision: 1.0–8.9%. Interday accuracy: 96.7%–104.0%.	Nicotinic acid: 2.0 (prehidrolysis) and 56.2 (after hydrolysis) μM.	[66]
Benzoic (BA), phenylpropionic (PhPA), phenyllactic (PhLA), 4-hydroxybenzoic (*p*-HBA), 4-hydroxyphenylacetic (*p*-HPhAA), 4-hydroxyphenylpropionic (*p*-HPhPA), homovanillic (HVA), 4-hydroxyphenyllactic (*p*-HPhLA).	GC–MS: 30 m × 0.25 mm × 0.25 µm, TR-5ms.	CSF samples (*n* = 138) from neurosurgical patients (*n* = 84), pooled CSF for validation.	40 μL (MEPS) 200 μL (LLE).	MEPS: 40 µL CSF + 40 µL distilled water + MEPS + elution with diethyl ether + drying + 40 μL BSTFA, 30 min at 90 °С + cooling 30 min at 4 °С + 350 μL of hexane. LLE: 200 µL CSF + 800 µL distilled water + 0.3–0.5 g solid NaCl + 15 µL concentrated sulfuric acid + diethyl ether + extraction 2 × 1 mL + evaporation at 40 °С + derivatization as for MEPS.	Recovery: 40–90%. LOD: 0.1–0.3 µM. LOQ: 0.4–0.7 µM. Precision (the reproducibility, RSD): <20%. Accuracy (the relative error, RE): <±20%. Lin.: over 0.4–10 µM (*R*^2^ ≥ 0.99).	Median (BA/PhPA/PhLA/p-HBA/p-HPhAA/HVA/p-HPhLA), μM: 0.7/<LOQ/0.1/nd/<LOQ/0.3/0.7/2.5.	[67]
Guanidinoacetate, creatine	Stable isotope dilution GC–MS: SGE BPX-70.	GAMT-deficient patients (*n* = 8) and SLC6A8-deficient patients (*n* = 8)	100 μL	100 μL CSF + 50 μL NaHCO_3_ + 50 μL hexafluoroacetylacetone + 500 μL toluene + heating 2 h to 80 °C + 300 μL toluene phase + drying under N_2_ + 10 μL triethylamine + 100 μL 7% PFBBr in MeCN (*v*/*v*), 15 min + 200 μL 0.5N HCl + 1 mL hexane + extraction.	Linearity: 0.5–10 nmol and 0.05–0.5 nmol LOD (S/N = 5): 0.01 and 0.0012 μM. LOQ (S/N = 10): 0.02 and 0.0024 μM. Intra-assay (*n* = 10): 0.25 ± 0.02 (CV 6.0%) and 57 ± 3 (CV 6.0%) μM. Interassay (*n* = 5): 0.25 ± 0.01(CV 4.0%) and 62 ± 3.7(CV 6.0%) μM	Control (*n* = 25): 0.036—0.22 μM and 24–66 μM GAMT deficient: 14–15 μM and not detected SLC6A8 deficient creatine levels: 56–62 μM.	[63]
Gamma-hydroxybutyric acid (GHB)	GC–MS: 30 m × 0.25 mm × 0.25 µm, VF-5 ms.	From autopsy cases (*n* = 21)	50 µL	50 μL CSF + IS + 200 μL 0.1M HCl + 1 mL ethyl acetate + centrifugation + evaporation to dryness at 20 °C, 2 mbar in a vacuum centrifuge + 50 μL MeCN + 25 μL BSTFA+1% TMCS + mixing.	Inter- and intraday accuracy: ≥91%. Imprecision: ≤9%. LOD: 0.5 mg/L. LOQ: 0.6 mg/L. Cal. curve: 1.0 mg/L, 10 mg/L, 40 mg/L, 80 mg/L and 100 mg/L.	Range concentrations after immediate analysis/after storage for 14 days at 4 °C/20 °C, mg/L: 1.1–10.4/0.6–13.2/<0.5–21.6.	[68]
Gamma-aminobutyric acid (GABA)	Isotope-dilution GC–ECNI-MS: 25 m × 0.32 mm, CPSil 88.	CSF samples of a patient before and during Vigabatrin treatment, control samples.	50, 500 µL	Free GABA: 500 μL CSF + 800 μL 1M phosphate buffer, pH 11.5 + 50 μL methylchloroformate + 150 μL 6 M HCl + 4 mL ethyl acetate + drying under N_2_ at 40 °C + 100 μL 7% PFBBr in MeCN + 10 μL triethylamine + 150 μL 0.5 M HCl + 1 mL hexane + drying under N_2_ at 40 °C + 50 μL hexane. Total GABA: 50 μL CSF + 450 μL water + 250 μL 20% sulphosalicylic acid + hydrolysis 24 h at 110 °C.	LOD: <0.005 µM. Free GABA. Intra-assay: 0.188 ± 0.004 µM (1.9% SD). Interassay: 0.177 ± 0.013 µM (7.3% SD). Total GABA. Intra-assay: 3.00 ± 0.05 µM (1.8% SD). Interassay: 3.57 ± 0.33 µM (9.2% SD).	Free/total GABA, µM. Control: 0.029–0.127/4.72–11.8. Before therapy: 0.153/13.2. During therapy: 0.274/24.1.	[69]
Pipecolic acid	Isotope-dilution GC–ECNI-MS: 30 m × 0.25 mm, DB-19.	Pediatric CSF samples	500 µL	CSF/aqueous standard solution + 1.0 mL 1 M potassium phosphate–sodium carbonate buffer, pH 11.5 + 50 μL methyl chloroformate + 0.15 mL 6 M HCl, pH 2 + 4 mL ethyl acetate + drying under N_2_ + 5 μL PFBBr + 10 μL triethylamine in 50 μL MeCN + 1 mL hexane + washing with 0.5 mL 100 mM HCl + 50 μL hexane.	Lin. 0.05–5 nmol (*R* > 0.999). LOD: 1.6E-6 nmol of PA (~0.5 nM). Recovery (CV): 97.3%–101.2% (4.0%–7.2%)	Patients: 0.93–4.53 µM. Control: 0.010–0.120 µM.	[70]
Androsterone, dihydrotestosterone, testosterone, allopregnanolone, isopregnanolone, pregnenolone.	GC–ECNI-MS: 15 m × 0.25 mm × 0.05 µm, HP 5890.	Normal volunteers, cisternal monkey.	1–2 mL	1–2 mL CSF + 100 mg C18 SPE + 50 μL 0.2% carboxymethoxylamine hemihydrochloride in pyridine + incubation 45 min at 60 °C + drying in N_2_+ 100 μL 1.25% pentafluorobenzyl bromide + 2.5% di-isopropylethylamine in MeCN + drying in N_2_ + 100 μL 50% BSTFA in MeCN + drying + 5 μL hexane.	Recovery: 78.2–99.5%. Reproducibility (RSD): 4.6–35.0%. Lin.: 10–1000 pg/ml (*R*^2^ > 0.996). Two-month variation <10%.	Human/monkey CSF, pg/mL: androsterone—52.8/24.7; testosterone—158.3/73.7; allopregnanolone—44.1/6.3; pregnenolone—52.8/16.7.	[71]
Pregnenolone, dehydroepiandrosterone, progesterone, androstenedione, testosterone, allopregnanolone, isopregnanolone, androsterone, epiandrosterone, 7α-hydroxy-dehydroepiandrosterone, 7β-Hydroxy-dehydroepiandrosterone, 5-androstene-3β,7α,17β-triol, 5-androstene-3β,7β,17β-triol, 16α-hydroxy-pregnenolone, 16α-hydroxy-dehydroepiandrosterone, 16α-hydroxy-progesterone.	GC–MS: 15 m × 0.25 mm × 0.1 μm, RESTEK Rxi.	Patients that underwent an endoscopic third ventriculostomy because of obstructive hydrocephalus (*n* = 15).	1 mL	1 mL CSF + 3 mL of diethyl ether + drying at 37 °C + 1 mL MeOH-water (4:1) + 1 mL pentane + drying of the polar phase 2 h in the vacuum centrifuge at 60 °C + 50 µL methoxylamine-hydrochloride solution in pyridine (2%) + incubation 1 h at 60 °C + drying in the N_2_ + 50 µL Sylon B + incubating 1 h at 90 °C + drying in the N_2_ + 20 µL isooctane.	Lin.: 10–1000 pg Slope: 0.96–1.33. *R*: >0.995. CV: 1.0–5.1%. LOD: 0.04–11.3 pM. Recovery: 75–104%.	Median, nM: 0.060/0.078/0.235/0.208/0.231/0.008/0.040/0.005/0.004/0.300/0.037/0.007/0.012/0.001/0.006/0.072.	[72]
Indomethacin	GC–NICI-MS: 30 m × 0.25 mm × 0.25 μm, HP-5MS.	Children (*n* = 31).	250 μL	250 μL CSF + acidification + C18 SPE + evaporation + 200 μL PFBBr (3.5% *v*/*v*, in MeCN) + 50 μL di-isopropylethylamine + extraction (water and toluene).	LOQ: 0.1 ng/sample. Accuracy: 98–122%. Recovery: 85–87%. Intraday (RSD, *n* = 3): 3–34%. Lin.: 0.1–5 ng/sample.	0.2–5.0 ng/mL (median, 1.4 ng/mL)	[73]
Scyllo-Inositol (Elnd005)	GC–MS	Healthy adults (*n* = 8).	No data	Extraction from human CSF diluted 1:1 with blank human plasma by protein precipitation/derivatization/LLE.	LLOQ: 0.4 µg/mL. Linearity 0.4–80 µg/mL. Precision for QC samples: 1.7–2.3%. Accuracy at all concentrations: 0.5% to +3.0%.	Prior to administration of ELND005 1.4–1.5 µg/mL.	[74]
6-monoacetyl morphine, morphine, codeine	GC–MS: 30 m × 0.25 mm × 0.25 µm, HP-5.	Deceased individuals (*n* = 25).	3 mL	3 mL CSF + 18 mL 0.1M phosphate buffer, pH 6.0 + SPE + 100 μL toluene + analysis of codeine + toluene evaporation + 50 μL BSTFA + 1% TMCS 10 min at 70 °C.	Linearity: to 1.7 mg/L. LOQ: 0.002 mg/L for morphine and 0.001 mg/L for codeine and 6-monoacetyl morphine. Precision: 0.115–0.121 mg/L (CV 4.4–7.2%).	0.001–0.406/0.01–0.38/<0.01–0.04 mg/L	[75]
Diethylene glycol, ethylene glycol, glycolic, oxalic, diglycolic, hydroxyethoxy acetic acids.	GC–NICI-MS: 30 m × 0.25 mm ID × 0.50 μm, ZB-5 ms.	Control CSF	250 µL	250 µL CSF + 1.0 mL water + 100 µL 5 N NaOH + 500 µL toluene + 50 µL PFBCl	LOQ: 0.05–1.0 µg/mL. Lin.: for diethylene glycol and ethylene glycol 0.02–2 (*R*^2^ 0.9984–0.9989) µg/mL; for other 0.05–51 (0.9990), 0.5–25 (0.9907), 0.5–100 (0.9985), 1–100 (0.9922) µg/mL. Accuracy: ≤15%.	No data	[76]

## Abbreviations

APCI	atmospheric pressure chemical ionization
BA	benzoic acid
BBB	blood-brain barrier
BSTFA	*N, O*-bis(trimethylsilyl)trifluoroacetamide
CI	chemical ionization
CNS	central nervous system
CSF	cerebrospinal fluid
CV	coefficient of variation
DLLME	dispersive liquid–liquid microextraction
dSPE	dispersive solid-phase extraction
ECNI	electron-capture negative-ion chemical ionization
EMA	European Medicines Agency
FDA	Food and Drug Administration
FTMS	Fourier transform mass spectrometry
F_2_-IsoPs	F_2_-isoprostanes
F_4_-NPs	F_4_-neuroprotanes
GABA	gamma-aminobutyric acid
GAMT	guanidinoacetate methyltransferase
GC	gas chromatography
GHB	gamma-hydroxybutyric acid
HEPES	4-(2-hydroxyethyl)-1-piperazineethanesulfonic acid
HIV	human immunodeficiency virus
HLLME	homogenous liquid–liquid microextraction
HPLC	high-performance liquid chromatography
5-HT	5-hydroxytryptamine
5-HTP	5-hydroxytryptophan
HVA	homovanillic acid
5-HIAA	5-hydroxyindole-3-acetic acid
5-HTOL	5-hydroxyindole-3-ethanol
ICP	inductively coupled plasma
IS	internal standard
3IAA	indole-3-acetic acid
3ICA	indole-3-carboxylic acid
3ILA	indole-3-lactic acid
3IPA	indole-3-propionic acid
LC	liquid chromatography
LLE	Liquid–liquid extraction
LLOD	lower limit of detection
LLOQ	lower limit of quantitation
LOD	limit of detection
LOQ	limit of quantitation
MALDI	matrix-assisted laser desorption/ionization
ME	matrix effect
MEPS	microextraction by packed sorbent
MS	mass spectrometry
MS/MS	tandem mass spectrometry
MSn	multistage mass spectrometry
MSTFA	*N*-methyl-*N*-(trimethylsilyl)trifluoroacetamide
MTBSTFA	*N*-(*tert*-butyldimethylsilyl)-*N*-methyltrifluoroacetamide
NMR	nuclear magnetic resonance
PFBBr	2,3,4,5,6-pentafluorobenzyl bromide
PFBCl	2,3,4,5,6-pentafluorobenzoyl chloride
p-HBA	4-hydroxybenzoic acid
PhLA	phenyllactic acid
PhPA	phenylpropionic acid
p-HPhAA	4-hydroxyphenylacetic acid
p-HphLA	4-hydroxyphenyllactic acid
p-HphPA	4-hydroxyphenylpropionic acid
PTV	programmed temperature vaporizer
Q	single quardupole
QuEChERS	“quick, easy, cheap, effective, rugged, and safe”
RE	relative error
RSD	relative standard deviation
SID	stable isotope dilution
SPE	solid-phase extraction
SPME	solid-phase microextraction
TBDMS	*tert*-butyldimethylsilyl
TMCS	trimethylchlorosilane
TOF	time-of-flight

## References

[1] Segal M.B. (1993). Extracellular and cerebrospinal fluids. J. Inherit. Metab. Dis..

[2] Noga M.J., Dane A., Shi S., Attali A., van Aken H., Suidgeest E., Tuinstra T., Muilwijk B., Coulier L., Luider T. (2012). Metabolomics of cerebrospinal fluid reveals changes in the central nervous system metabolism in a rat model of multiple sclerosis. Metabolomics.

[3] Regenold W.T., Phatak P., Makley M.J., Stone R.D., Kling M.A. (2008). Cerebrospinal fluid evidence of increased extra-mitochondrial glucose metabolism implicates mitochondrial dysfunction in multiple sclerosis disease progression. J. Neurol. Sci..

[4] Obeid R., Kasoha M., Knapp J.P., Kostopoulos P., Becker G., Fassbender K., Herrmann W. (2007). Folate and methylation status in relation to phosphorylated tau protein(181P) and β-amyloid(1-42) in cerebrospinal fluid. Clin. Chem..

[5] Jasperse B., Jakobs C., Eikelenboom M.J., Dijkstra C.D., Uitdehaag B.M.J., Barkhof F., Polman C.H., Teunissen C.E. (2007). N-acetylaspartic acid in cerebrospinal fluid of multiple sclerosis patients determined by gas-chromatography-mass spectrometry. J. Neurol..

[6] Mattsson N., Yaong M., Rosengren L., Blennow K., Månsson J.-E., Andersen O., Zetterberg H., Haghighi S., Zho I., Pratico D. (2009). Elevated cerebrospinal fluid levels of prostaglandin E2 and 15-(S)-hydroxyeicosatetraenoic acid in multiple sclerosis. J. Intern. Med..

[7] Lim C.K., Bilgin A., Lovejoy D.B., Tan V., Bustamante S., Taylor B.V., Bessede A., Brew B.J., Guillemin G.J. (2017). Kynurenine pathway metabolomics predicts and provides mechanistic insight into multiple sclerosis progression. Sci. Rep..

[8] Lewitt P.A., Li J., Lu M., Guo L., Auinger P. (2017). Metabolomic biomarkers as strong correlates of Parkinson disease progression. Neurology.

[9] Naylor J.C., Hulette C.M., Steffens D.C., Shampine L.J., Ervin J.F., Payne V.M., Massing M.W., Kilts J.D., Strauss J.L., Calhoun P.S. (2008). Cerebrospinal fluid dehydroepiandrosterone levels are correlated with brain dehydroepiandrosterone levels, elevated in Alzheimer’s disease, and related to neuropathological disease stage. J. Clin. Endocrinol. Metab..

[10] Fonteh A.N., Cipolla M., Chiang J., Arakaki X., Harrington M.G. (2014). Human cerebrospinal fluid fatty acid levels differ between supernatant fluid and brain-derived nanoparticle fractions, and are altered in Alzheimer’s disease. PLoS ONE.

[11] Praticò D., Clark C.M., Liun F., Lee V.Y.M., Trojanowski J.Q. (2002). Increase of Brain Oxidative Stress in Mild Cognitive Impairment. Arch. Neurol..

[12] Yao Y., Clark C.M., Trojanowski J.Q., Lee V.M.Y., Praticò D. (2005). Elevation of 12/15 lipoxygenase products in AD and mild cognitive impairment. Ann. Neurol..

[13] Fonteh A.N., Cipolla M., Chiang A.J., Edminster S.P., Arakaki X., Harrington M.G. (2020). Polyunsaturated Fatty Acid Composition of Cerebrospinal Fluid Fractions Shows Their Contribution to Cognitive Resilience of a Pre-symptomatic Alzheimer’s Disease Cohort. Front. Physiol..

[14] Montine T.J., Kaye J.A., Montine K.S., McFarland L., Morrow J.D., Quinn J.F. (2001). Cerebrospinal fluid Aβ42, tau, and F2-isoprostane concentrations in patients with Alzheimer disease, other dementias, and in age-matched controls. Arch. Pathol. Lab. Med..

[15] Chernevskaya E., Beloborodova N., Klimenko N., Pautova A., Shilkin D., Gusarov V., Tyakht A., Tyakht A. (2020). Serum and fecal profiles of aromatic microbial metabolites reflect gut microbiota disruption in critically ill patients: A prospective observational pilot study. Crit. Care.

[16] Chernevskaya E., Klimenko N., Pautova A., Buyakova I., Tyakht A., Beloborodova N. (2021). Host-microbiome interactions mediated by phenolic metabolites in chronically critically ill patients. Metabolites.

[17] Carabotti M., Scirocco A., Maselli M.A., Severi C. (2015). The gut-brain axis: Interactions between enteric microbiota, central and enteric nervous systems. Ann. Gastroenterol..

[18] Kuwahara A., Matsuda K., Kuwahara Y., Asano S., Inui T., Marunaka Y. (2020). Microbiota-gut-brain axis: Enteroendocrine cells and the enteric nervous system form an interface between the microbiota and the central nervous system. Biomed. Res..

[19] Chen M.X., Wang S.Y., Kuo C.H., Tsai I.L. (2019). Metabolome analysis for investigating host-gut microbiota interactions. J. Formos. Med. Assoc..

[20] Doherty C.M., Forbes R.B. (2014). Diagnostic lumbar puncture. Ulster Med. J..

[21] Wang L.P., Schmidt J.F. (1997). Central nervous side effects after lumbar puncture. A review of the possible pathogenesis of the syndrome of postdural puncture headache and associated symptoms. Dan. Med. Bull..

[22] Wishart D.S., Lewis M.J., Morrissey J.A., Flegel M.D., Jeroncic K., Xiong Y., Cheng D., Eisner R., Gautam B., Tzur D. (2008). The human cerebrospinal fluid metabolome. J. Chromatogr. B Anal. Technol. Biomed. Life Sci..

[23] Nakamizo S., Sasayama T., Shinohara M., Irino Y., Nishiumi S., Nishihara M., Tanaka H., Tanaka K., Mizukawa K., Itoh T. (2013). GC/MS-based metabolomic analysis of cerebrospinal fluid (CSF) from glioma patients. J. Neurooncol..

[24] Murgia F., Lorefice L., Poddighe S., Fenu G., Secci M.A., Marrosu M.G., Cocco E., Atzori L. (2020). Multi-Platform Characterization of Cerebrospinal Fluid and Serum Metabolome of Patients Affected by Relapsing–Remitting and Primary Progressive Multiple Sclerosis. J. Clin. Med..

[25] Wuolikainen A., Moritz T., Marklund S.L., Antti H., Andersen P.M. (2011). Disease-Related Changes in the Cerebrospinal Fluid Metabolome in Amyotrophic Lateral Sclerosis Detected by GC/TOFMS. PLoS ONE.

[26] Park S.J., Jeong I.H., Kong B.S., Lee J.E., Kim K.H., Lee D.Y., Kim H.J. (2016). Disease type- and status-specific alteration of CSF metabolome coordinated with clinical parameters in inflammatory demyelinating diseases of CNS. PLoS ONE.

[27] Gordon S.M., Srinivasan L., Taylor D.M., Master S.R., Tremoglie M.A., Hankeova A., Flannery D.D., Abbasi S., Fitzgerald J.C., Harris M.C. (2020). Derivation of a metabolic signature associated with bacterial meningitis in infants. Pediatr. Res..

[28] Kraus V.B. (2018). Biomarkers as drug development tools: Discovery, validation, qualification and use. Nat. Rev. Rheumatol..

[29] Koek M.M., Jellema R.H., van der Greef J., Tas A.C., Hankemeier T. (2011). Quantitative metabolomics based on gas chromatography mass spectrometry: Status and perspectives. Metabolomics.

[30] Mandal R., Guo A.C., Chaudhary K.K., Liu P., Yallou F.S., Dong E., Aziat F., Wishart D.S. (2012). Multi-platform characterization of the human cerebrospinal fluid metabolome: A comprehensive and quantitative update. Genome Med..

[31] Stoop M.P., Coulier L., Rosenling T., Shi S., Smolinska A.M., Buydens L., Ampt K., Stingl C., Dane A., Muilwijk B. (2010). Quantitative proteomics and metabolomics analysis of normal human cerebrospinal fluid samples. Mol. Cell. Proteomics.

[32] Saito K., Hattori K., Andou T., Satomi Y., Gotou M., Kobayashi H., Hidese S., Kunugi H. (2020). Characterization of postprandial effects on csf metabolomics: A pilot study with parallel comparison to plasma. Metabolites.

[33] Rosenling T., Slim C.L., Christin C., Coulier L., Shi S., Stoop M.P., Bosman J., Suits F., Horvatovich P.L., Stockhofe-Zurwieden N. (2009). The Effect of Preanalytical Factors on Stability of the Proteome and Selected Metabolites in Cerebrospinal Fluid (CSF). J. Proteome Res..

[34] Hartonen M., Mattila I., Ruskeepää A.L., Orešič M., Hyötyläinen T. (2013). Characterization of cerebrospinal fluid by comprehensive two-dimensional gas chromatography coupled to time-of-flight mass spectrometry. J. Chromatogr. A.

[35] Carrasco-Pancorbo A., Nevedomskaya E., Arthen-Engeland T., Zey T., Zurek G., Baessmann C., Deelder A.M., Mayboroda O.A. (2009). Gas chromatography/atmospheric pressure chemical ionization-time of flight mass spectrometry: Analytical validation and applicability to metabolic profiling. Anal. Chem..

[36] Akiyama T., Saigusa D., Hyodo Y., Umeda K., Saijo R., Koshiba S., Kobayashi K. (2020). Metabolic Profiling of the Cerebrospinal Fluid in Pediatric Epilepsy. Acta Med. Okayama.

[37] Li C., Xu F., Xie D.M., Jing Y., Shang M.Y., Liu G.X., Wang X., Cai S.Q. (2014). Identification of absorbed constituents in the rabbit plasma and cerebrospinal fluid after intranasal administration of asari radix et rhizoma by HS-SPME-GC-MS and HPLC-APCI-IT-TOF-MSN. Molecules.

[38] Deng F.L., Pan J.X., Zheng P., Xia J.J., Yin B.M., Liang W.W., Li Y.F., Wu J., Xu F., Wu Q.Y. (2019). Metabonomics reveals peripheral and central shortchain fatty acid and amino acid dysfunction in a naturally occurring depressive model of macaques. Neuropsychiatr. Dis. Treat..

[39] Coulier L., Muilwijk B., Bijlsma S., Noga M., Tienstra M., Attali A., van Aken H., Suidgeest E., Tuinstra T., Luider T.M. (2013). Metabolite profiling of small cerebrospinal fluid sample volumes with gas chromatography–mass spectrometry: Application to a rat model of multiple sclerosis. Metabolomics.

[40] Koek M.M., Bakels F., Engel W., Van Den Maagdenberg A., Ferrari M.D., Coulier L., Hankemeier T. (2010). Metabolic profiling of ultrasmall sample volumes with GC/MS: From microliter to nanoliter samples. Anal. Chem..

[41] Lu A.Y., Damisah E.C., Winkler E.A., Grant R.A., Eid T., Bulsara K.R. (2018). Cerebrospinal fluid untargeted metabolomic profiling of aneurysmal subarachnoid hemorrhage: An exploratory study. Br. J. Neurosurg..

[42] Orefice N.S., Carotenuto A., Mangone G., Bues B., Rehm R., Cerillo I., Saccà F., Calignano A., Orefice G. (2016). Assessment of neuroactive steroids in cerebrospinal fluid comparing acute relapse and stable disease in relapsing-remitting multiple sclerosis. J. Steroid Biochem. Mol. Biol..

[43] Di Filippo M., Pini L.A., Pelliccioli G.P., Calabresi P., Sarchielli P. (2008). Abnormalities in the cerebrospinal fluid levels of endocannabinoids in multiple sclerosis. J. Neurol. Neurosurg. Psychiatry.

[44] Paik M.J., Ahn Y.H., Lee P.H., Kang H., Park C.B., Choi S., Lee G. (2010). Polyamine patterns in the cerebrospinal fluid of patients with Parkinson’s disease and multiple system atrophy. Clin. Chim. Acta.

[45] Lee P.H., Lee G., Paik M.J. (2008). Polyunsaturated fatty acid levels in the cerebrospinal fluid of patients with Parkinson’s disease and multiple system atrophy. Mov. Disord..

[46] Zhang P., Wang B., Sun Y., Gao J., Lian K. (2020). Analysis of 5-hydroxytryptamine and its related indoles in cerebrospinal fluid of leukemic children by gas chromatography-mass spectrometry. J. Lab. Med..

[47] Mason S., Reinecke C.J., Solomons R. (2017). Cerebrospinal Fluid Amino Acid Profiling of Pediatric Cases with Tuberculous Meningitis. Front. Neurosci..

[48] Obata T., Nagakura T., Maeda H., Yamashita K., Maekawa K. (1999). Simultaneous assay of prostaglandins and thromboxane in the cerebrospinal fluid by gas chromatography-mass spectrometry-selected ion monitoring. J. Chromatogr. B Biomed. Sci. Appl..

[49] Kozar M.P., Krahmer M.T., Fox A., Gray B.M. (2000). Failure to detect muramic acid in normal rat tissues but detection in cerebrospinal fluids from patients with pneumococcal meningitis. Infect. Immun..

[50] Medana I.M., Hien T.T., Day N.P., Phu N.H., Mai N.T.H., Van Chu’ong L., Chau T.T.H., Taylor A., Salahifar H., Stocker R. (2002). The Clinical Significance of Cerebrospinal Fluid Levels of Kynurenine Pathway Metabolites and Lactate in Severe Malaria. J. Infect. Dis..

[51] Medana I.M., Day N.P.J., Salahifar-Sabet H., Stocker R., Smythe G., Bwanaisa L., Njobvu A., Kayira K., Turner G.D.H., Taylor T.E. (2003). Metabolites of the kynurenine pathway of tryptophan metabolism in the cerebrospinal fluid of Malawian children with malaria. J. Infect. Dis..

[52] Anderson A.M., Croteau D., Ellis R.J., Rosario D., Potter M., Guillemin G.J., Brew B.J., Woods S.P., Letendre S.L. (2018). HIV, prospective memory, and cerebrospinal fluid concentrations of quinolinic acid and phosphorylated Tau. J. Neuroimmunol..

[53] Inoue H., Matsushige T., Ichiyama T., Okuno A., Takikawa O., Tomonaga S., Anlar B., Yüksel D., Otsuka Y., Kohno F. (2020). Elevated quinolinic acid levels in cerebrospinal fluid in subacute sclerosing panencephalitis. J. Neuroimmunol..

[54] Regenold W.T., Kling M.A., Hauser P. (2000). Elevated sorbitol concentration in the cerebrospinal fluid of patients with mood disorders. Psychoneuroendocrinology.

[55] Kageyama Y., Deguchi Y., Hattori K., Yoshida S., Goto Y., Inoue K., Kato T. (2021). Nervonic acid level in cerebrospinal fluid is a candidate biomarker for depressive and manic symptoms: A pilot study. Brain Behav..

[56] Kim B.K., Fonda J.R., Hauger R.L., Pinna G., Anderson G.M., Valovski I.T., Rasmusson A.M. (2020). Composite contributions of cerebrospinal fluid GABAergic neurosteroids, neuropeptide Y and interleukin-6 to PTSD symptom severity in men with PTSD. Neurobiol. Stress.

[57] Rasmusson A.M., King M.W., Valovski I., Gregor K., Scioli-Salter E., Pineles S.L., Hamouda M., Nillni Y.I., Anderson G.M., Pinna G. (2019). Relationships between cerebrospinal fluid GABAergic neurosteroid levels and symptom severity in men with PTSD. Psychoneuroendocrinology.

[58] Jokinen J., Nordström A.L., Nordström P. (2009). Cerebrospinal fluid monoamine metabolites and suicide. Nord. J. Psychiatry.

[59] Engström G., Alling C., Blennow K., Regnéll G., Träskman-Bendz L. (1999). Reduced cerebrospinal HVA concentrations and HVA/5-HIAA ratios in suicide attempters: Monoamine metabolites in 120 suicide attempters and 47 controls. Eur. Neuropsychopharmacol..

[60] Lindqvist D., Janelidze S., Hagell P., Erhardt S., Samuelsson M., Minthon L., Hansson O., Björkqvist M., Träskman-Bendz L., Brundin L. (2009). Interleukin-6 Is Elevated in the Cerebrospinal Fluid of Suicide Attempters and Related to Symptom Severity. Biol. Psychiatry.

[61] Mochel F., DeLonlay P., Touati G., Brunengraber H., Kinman R.P., Rabier D., Roe C.R., Saudubray J.M. (2005). Pyruvate carboxylase deficiency: Clinical and biochemical response to anaplerotic diet therapy. Mol. Genet. Metab..

[62] Mazzuca M., Maubert M.A., Damaj L., Clot F., Cadoudal M., Dubourg C., Odent S., Benoit J.F., Bahi-Buisson N., Christa L. (2015). Combined Sepiapterin Reductase and Methylmalonyl-CoA Epimerase Deficiency in a Second Patient: Cerebrospinal Fluid Polyunsaturated Fatty Acid Level and Follow-Up Under L-DOPA, 5-HTP and BH4 Trials. JIMD Rep..

[63] Almeida L.S., Verhoeven N.M., Roos B., Valongo C., Cardoso M.L., Vilarinho L., Salomons G.S., Jakobs C. (2004). Creatine and guanidinoacetate: Diagnostic markers for inborn errors in creatine biosynthesis and transport. Mol. Genet. Metab..

[64] de Paiva M.J.N., Menezes H.C., Christo P.P., Resende R.R., Cardeal Z.d.L. (2013). An alternative derivatization method for the analysis of amino acids in cerebrospinal fluid by gas chromatography-mass spectrometry. J. Chromatogr. B Anal. Technol. Biomed. Life Sci..

[65] Pautova A., Khesina Z., Getsina M., Sobolev P., Revelsky A., Beloborodova N. (2020). Determination of Tryptophan Metabolites in Serum and Cerebrospinal Fluid Samples Using Microextraction by Packed Sorbent, Silylation and GC-MS Detection. Molecules.

[66] Smythe G.A., Braga O., Brew B.J., Grant R.S., Guillemin G.J., Kerr S.J., Walker D.W. (2002). Concurrent Quantification of Quinolinic, Picolinic, and Nicotinic Acids Using Electron-Capture Negative-Ion Gas Chromatography–Mass Spectrometry. Anal. Biochem..

[67] Pautova A.K., Khesina Z.B., Litvinova T.N., Revelsky A.I., Beloborodova N.V. (2021). Metabolic profiling of aromatic compounds in cerebrospinal fluid of neurosurgical patients using microextraction by packed sorbent and liquid–liquid extraction with gas chromatography–mass spectrometry analysis. Biomed. Chromatogr..

[68] Kietzerow J., Otto B., Wilke N., Rohde H., Iwersen-Bergmann S., Andresen-Streichert H. (2020). The challenge of post-mortem GHB analysis: Storage conditions and specimen types are both important. Int. J. Legal Med..

[69] Kok R.M., Howells D.W., Van Den Heuvel C.C.M., Guérand W.S., Thompson G.N., Jakobs C. (1993). Stable Isotope Dilution Analysis of GABA in CSF Using Simple Solvent Extraction and Electron-Capture Negative-Ion Mass Fragmentography. J. Inherit. Metab. Dis..

[70] Kok R.M., Kaster L., De Jong A.P., Poll-Thé B., Saudubray J.-M., Jakobs C. (1987). Stable isotope dilution analysis of pipecolic acid in cerebrospinal fluid, plasma, urine and amniotic fluid using electron capture negative ion mass fragmentography. Clin. Chim. Acta..

[71] Kim Y.S., Zhang H., Kim H.Y. (2000). Profiling neurosteroids in cerebrospinal fluids and plasma by gas chromatography/electron capture negative chemical ionization mass spectrometry. Anal. Biochem..

[72] Kancheva R., Hill M., Novák Z., Chrastina J., Velíková M., Kancheva L., Říha I., Stárka L. (2010). Peripheral neuroactive steroids may be as good as the steroids in the cerebrospinal fluid for the diagnostics of CNS disturbances. J. Steroid Biochem. Mol. Biol..

[73] Mannila A., Kumpulainen E., Lehtonen M., Heikkinen M., Laisalmi M., Salo T., Rautio J., Savolainen J., Kokki H. (2007). Plasma and cerebrospinal fluid concentrations of indomethacin in children after intravenous administration. J. Clin. Pharmacol..

[74] Liang E., Garzone P., Cedarbaum J.M., Koller M., Tran T., Xu V., Ross B., Jhee S.S., Ereshefsky L., Pastrak A. (2013). Pharmacokinetic profile of orally administered scyllo-inositol (Elnd005) in plasma, cerebrospinal fluid and brain, and corresponding effect on amyloid-beta in healthy subjects. Clin. Pharmacol. Drug Dev..

[75] Wyman J., Bultman S. (2004). Postmortem Distribution of Heroin Metabolites in Femoral Blood, Liver, Cerebrospinal Fluid, and Vitreous Humor. J. Anal. Toxicol..

[76] Perala A.W., Filary M.J., Bartels M.J., McMartin K.E. (2014). Quantitation of Diethylene Glycol and Its Metabolites by Gas Chromatography Mass Spectrometry or Ion Chromatography Mass Spectrometry in Rat and Human Biological Samples. J. Anal. Toxicol..

[77] Wardlaw S.L., Burant C.F., Klein S., Meece K., White A., Kasten T., Lucey B.P., Bateman R.J. (2014). Continuous 24-hour leptin, proopiomelanocortin, and amino acid measurements in human cerebrospinal fluid: Correlations with plasma leptin, soluble leptin receptor, and amino acid levels. J. Clin. Endocrinol. Metab..

[78] Young S., Struys E., Wood T. (2007). Quantification of creatine and guanidinoacetate using GC-MS and LC-MS/MS for the detection of cerebral creatine deficiency syndromes. Curr. Protoc. Hum. Genet..

[79] Plecko B., Paul K., Paschke E., Stoeckler-Ipsiroglu S., Struys E., Jakobs C., Hartmann H., Luecke T., di Capua M., Korenke C. (2007). Biochemical and Molecular Characterizationof 18 Patients With Pyridoxine-Dependent Epilepsyand Mutations of the Antiquitin (ALDH7A1) Gene. Hum. Mutat..

[80] Wang Y., Karu K., Griffiths W.J. (2007). Analysis of neurosterols and neurosteroids by mass spectrometry. Biochimie.

[81] Starka L., Hill M., Kancheva R., Novak Z., Chrastina J., Pohanka M., Morfin R. (2009). 7-Hydroxylated derivatives of dehydroepiandrosterone in the human ventricular cerebrospinal fluid. Neuroendocrinol. Lett..

[82] Miller E., Morel A., Saso L., Saluk J. (2014). Isoprostanes and neuroprostanes as biomarkers of oxidative stress in neurodegenerative diseases. Oxid. Med. Cell. Longev..

[83] Praticò D., Clark C.M., Lee V.M.-Y., Trojanowski J.Q., Rokach J., FitzGerald G.A. (2000). Increased 8,12-iso-iPF 2-VI in Alzheimer’s Disease: Correlation of a Noninvasive Index of Lipid Peroxidation with Disease Severity. Ann. Neurol..

[84] Li G., Millard S.P., Peskind E.R., Zhang J., Yu C.E., Leverenz J.B., Mayer C., Shofer J.S., Raskind M.A., Quinn J.F. (2014). Cross-sectional and longitudinal relationships between cerebrospinal fluid biomarkers and cognitive function in people without cognitive impairment from across the adult life span. JAMA Neurol..

[85] Finno C.J., Estell K.E., Winfield L., Katzman S., Bordbari M.H., Burns E.N., Miller A.D., Puschner B., Tran C.K., Xu L. (2018). Lipid peroxidation biomarkers for evaluating oxidative stress in equine neuroaxonal dystrophy. J. Vet. Intern. Med..

[86] Yen H.C., Wei H.J., Chen T.W. (2013). Analytical variables affecting analysis of Fisoprostanes and Fneuroprostanes in human cerebrospinal fluid by gas chromatography/mass spectrometry. Biomed. Res. Int..

[87] Milne G.L., Sanchez S.C., Musiek E.S., Morrow J.D. (2007). Quantification of F2-isoprostanes as a biomarker of oxidative stress. Nat. Protoc..

[88] Dmitrienko S.G., Apyari V.V., Gorbunova M.V., Tolmacheva V.V., Zolotov Y.A. (2020). Homogeneous Liquid–Liquid Microextraction of Organic Compounds. J. Anal. Chem..

[89] Gupta M., Jain A., Verma K.K. (2009). Salt-assisted liquid-liquid microextraction with water-miscible organic solvents for the determination of carbonyl compounds by high-performance liquid chromatography. Talanta.

[90] Rezaee M., Assadi Y., Milani Hosseini M.R., Aghaee E., Ahmadi F., Berijani S. (2006). Determination of organic compounds in water using dispersive liquid-liquid microextraction. J. Chromatogr. A.

[91] Dmitrienko S.G., Apyari V.V., Tolmacheva V.V., Gorbunova M.V. (2020). Dispersive Liquid–Liquid Microextraction of Organic Compounds: An Overview of Reviews. J. Anal. Chem..

[92] Zuloaga O., Olivares M., Navarro P., Vallejo A., Prieto A. (2015). Dispersive liquid-liquid microextraction: Trends in the analysis of biological samples. Bioanalysis.

[93] Mudiam M.K.R., Ratnasekhar C. (2013). Ultra sound assisted one step rapid derivatization and dispersive liquid-liquid microextraction followed by gas chromatography-mass spectrometric determination of amino acids in complex matrices. J. Chromatogr. A.

[94] Jain R., Kumar A., Shukla Y. (2015). Dispersive Liquid-liquid Microextraction-injector Port Silylation: A Viable Option for the Analysis of Polar Analytes using Gas Chromatography-Mass Spectrometry. Austin J. Anal. Pharm. Chem..

[95] Anastassiades M., Lehotay S.J., Štajnbaher D., Schenck F.J. (2003). Fast and easy multiresidue method employing acetonitrile extraction/partitioning and “dispersive solid-phase extraction” for the determination of pesticide residues in produce. J. AOAC Int..

[96] Townsend R., Keulen G., Desbrow C., Godfrey A.R. (2020). An investigation of the utility of QuEChERS for extracting acid, base, neutral and amphiphilic species from example environmental and clinical matrices. Anal. Sci. Adv..

[97] Rial-Berriel C., Acosta-Dacal A., Zumbado M., Luzardo O.P. (2020). Micro QuEChERS-based method for the simultaneous biomonitoring in whole blood of 360 toxicologically relevant pollutants for wildlife. Sci. Total Environ..

[98] Santana-Mayor Á., Socas-Rodríguez B., Herrera-Herrera A.V., Rodríguez-Delgado M.Á. (2019). Current trends in QuEChERS method. A versatile procedure for food, environmental and biological analysis. TrAC Trends Anal. Chem..

[99] Arthur C.L., Pawliszyn J. (1990). Solid Phase Microextraction with Thermal Desorption Using Fused Silica Optical Fibers. Anal. Chem..

[100] Vuckovic D., De Lannoy I., Gien B., Shirey R.E., Sidisky L.M., Dutta S., Pawliszyn J. (2011). In vivo solid-phase microextraction: Capturing the elusive portion of metabolome. Angew. Chemie Int. Ed..

[101] Campanella B., Onor M., Lomonaco T., Benedetti E., Bramanti E. (2019). HS-SPME-GC-MS approach for the analysis of volatile salivary metabolites and application in a case study for the indirect assessment of gut microbiota. Anal. Bioanal. Chem..

[102] Silva C.L., Passos M., Cmara J.S. (2011). Investigation of urinary volatile organic metabolites as potential cancer biomarkers by solid-phase microextraction in combination with gas chromatography-mass spectrometry. Br. J. Cancer.

[103] Tienpont B., David F., Desmet K., Sandra P. (2002). Stir bar sorptive extraction-thermal desorption-capillary GC-MS applied to biological fluids. Anal. Bioanal. Chem..

[104] Nazyropoulou C., Samanidou V. (2015). Stir bar sorptive extraction applied to the analysis of biological fluids. Bioanalysis.

[105] Hasan C.K., Ghiasvand A., Lewis T.W., Nesterenko P.N., Paull B. (2020). Recent advances in stir-bar sorptive extraction: Coatings, technical improvements, and applications. Anal. Chim. Acta.

[106] Li J., Qi H.Y., Wang Y.B., Su Q., Wu S., Wu L. (2016). Hollow fiber–stir bar sorptive extraction and microwave assisted derivatization of amino acids in biological matrices. J. Chromatogr. A.

[107] Farajzadeh M.A., Matin A.A. (2008). Determination of BTEX in water samples with an SPME hollow fiber coated copper wire. Chromatographia.

[108] Abdel-Rehim M. (2004). New trend in sample preparation: On-line microextraction in packed syringe for liquid and gas chromatography applications. J. Chromatogr. B.

[109] Pautova A.K., Sobolev P.D., Revelsky A.I. (2020). Microextraction of aromatic microbial metabolites by packed hypercrosslinked polystyrene from blood serum. J. Pharm. Biomed. Anal..

[110] Silva C., Cavaco C., Perestrelo R., Pereira J., Câmara J.S. (2014). Microextraction by packed Sorbent (MEPS) and solid-phase microextraction (SPME) as sample preparation procedures for the metabolomic profiling of urine. Metabolites.

[111] Peters S., Kaal E., Horsting I., Janssen H.G. (2012). An automated method for the analysis of phenolic acids in plasma based on ion-pairing micro-extraction coupled on-line to gas chromatography/mass spectrometry with in-liner derivatisation. J. Chromatogr. A.

[112] Bustamante L., Cárdenas D., von Baer D., Pastene E., Duran-Sandoval D., Vergara C., Mardones C. (2017). Evaluation of microextraction by packed sorbent, liquid–liquid microextraction and derivatization pretreatment of diet-derived phenolic acids in plasma by gas chromatography with triple quadrupole mass spectrometry. J. Sep. Sci..

[113] Pautova A.K., Sobolev P.D., Revelsky A.I. (2019). Analysis of phenylcarboxylic acid-type microbial metabolites by microextraction by packed sorbent from blood serum followed by GC–MS detection. Clin. Mass Spectrom..

